# Supplementary material for: Contributions of treatment centre and patient characteristics to patient-reported experience of haemodialysis: a national cross-sectional study
Source: BMJ Open. 2021 Apr 14;11(4):e044984. doi: 10.1136/bmjopen-2020-044984 (PMC8054084; doi:10.1136/bmjopen-2020-044984)
Supplement: Supplementary data [file bmjopen-2020-044984supp001.pdf]

The Annual Survey of Patient Reported Experience Measures (PREM)

The annual PREM survey has been designed by patients and professionals working together to find out how you feel about the services your kidney unit provides. The survey gives us feedback on renal services both locally for your unit, and nationally, and ensures that the views of kidney patients are heard. From this information, we can see what we are doing well and where we can do better. Your views matter and we act on them to help us improve services. We will provide you with feedback on the results. Information on the national results can be obtained from the UK Renal Registry or Kidney Care UK websites, along with the previous year's results.

The survey is completely anonymous, your name will not appear anywhere on the survey.

Completing the survey

The survey should take about 15 minutes to complete. Please only tick one box for each question or statement, otherwise your answer will not count.

If you prefer you can complete the survey online at www.renalreg.org/projects/prem. The online survey is available in English, Welsh, Urdu and Gujarati. Please only complete one paper PREM or one online, not both.

You can ask your partner, a friend or family member to help you complete the survey. Choosing not to take part will not affect your care in any way. When you complete the survey think about your experience of care during the last few times that you have attended. Please fill in the survey as truthfully as possible.

On completion

Please place the completed questionnaire in the envelope provided, seal it, and post it in the post box or hand it to a staff member. By completing the questionnaire you are consenting to your answers being sent to and held by the UK Renal Registry and your renal unit.

If you have any questions or concerns about the survey please contact the UK Renal Registry by emailing Catherine.stannard@renalregistry.nhs.uk or by calling 0117 414 8151.

Please complete the name of the renal/satellite unit where you are completing this survey from.

UKRR Code ☐☐☐☐☐☐☐ (To be filled in by a member of staff)

Renal Unit ☐☐☐☐☐☐☐☐☐☐☐☐☐☐☐☐☐☐☐☐

Current treatment

☐ Peritoneal dialysis ☐ Haemodialysis ☐ Transplant ☐ Attending kidney clinic but not on dialysis or transplantation

If you currently receive Haemodialysis, do you receive this…

☐ At Home ☐ In-Hospital ☐ In-Satellite

 Please turn over the page

Age ☐ 17-21 ☐ 22-30 ☐ 31-40 ☐ 41-55
☐ 56-64 ☐ 65-74 ☐ 75-84 ☐ 85+

Gender ☐ Male ☐ Female ☐ I would rather not say

Ethnicity ☐ Asian ☐ Black ☐ White ☐ Other ☐ I would rather not say

Do you use PatientView? ☐ Yes ☐ No ☐ Don't Know

The following questions ask you about your experience with the renal unit, your kidney disease and treatment.

All the questions are answered on a scale of 1 to 7 where 1 is negative and 7 is positive.

For each question there's also a 'don't know' and 'not applicable' option.

SECTION 1: ACCESS TO THE RENAL TEAM

	**Never** 1	2	3	4	5	6	**Always** 7	Don't know	Not Applicable
1. Does the renal team take time to answer your questions about your kidney disease or treatment?	☐	☐	☐	☐	☐	☐	☐	☐	☐
2. Would you feel comfortable to contact the unit from home if you were anxious or worried?	☐	☐	☐	☐	☐	☐	☐	☐	☐
3. Would you feel able to ask for an additional appointment with your kidney doctor if you wanted to?	☐	☐	☐	☐	☐	☐	☐	☐	☐

 Please turn over the page

SECTION 2: SUPPORT

Does the renal team help you to get the support you want with:

	Never 1	2	3	4	5	6	Always 7	Don't know	Not Applicable
4. Medical issues resulting from your kidney disease?	☐	☐	☐	☐	☐	☐	☐	☐	☐
5. Any other concerns or anxieties resulting from your kidney disease or treatment?	☐	☐	☐	☐	☐	☐	☐	☐	☐
6. Accessing patient support groups such as Kidney Patient Associations (KPA)?	☐	☐	☐	☐	☐	☐	☐	☐	☐

SECTION 3: COMMUNICATION

Do you think there is good communication between:

	Never 1	2	3	4	5	6	Always 7	Don't know	Not Applicable
7. You and your renal team?	☐	☐	☐	☐	☐	☐	☐	☐	☐
8. Members of the renal team?	☐	☐	☐	☐	☐	☐	☐	☐	☐
9. Your GP and the renal team?	☐	☐	☐	☐	☐	☐	☐	☐	☐
10. The renal team and other medical specialists?	☐	☐	☐	☐	☐	☐	☐	☐	☐
11. The renal team and other non-healthcare services if you need them, such as social work or housing?	☐	☐	☐	☐	☐	☐	☐	☐	☐

Please turn over the page

SECTION 4: PATIENT INFORMATION

Does the renal team:

	Never 1	2	3	4	5	6	Always 7	Don't know	Not Applicable
12. Explain things to you in a way that is easy to understand?	☐	☐	☐	☐	☐	☐	☐	☐	☐
13. Give you as much information about your kidney disease or treatment as you want?	☐	☐	☐	☐	☐	☐	☐	☐	☐

SECTION 5: FLUID INTAKE AND DIET

Thinking about the advice you are given about <u>fluid intake</u>:

	Never 1	2	3	4	5	6	Always 7	Don't know	Not Applicable
14. Does the renal team give you clear advice on your **fluid intake**?	☐	☐	☐	☐	☐	☐	☐	☐	☐

Thinking about the advice you are given about <u>diet</u>:

	Never 1	2	3	4	5	6	Always 7	Don't know	Not Applicable
15. Does the renal team give you clear advice on your **diet**?	☐	☐	☐	☐	☐	☐	☐	☐	☐

SECTION 6: NEEDLING

If you are on in-hospital or in-satellite haemodialysis please answer question 16, otherwise please go to SECTION 7: TESTS

	Never 1	2	3	4	5	6	Always 7	Don't know	Not Applicable
16. How often do the renal team insert your needles with as little pain as possible?	☐	☐	☐	☐	☐	☐	☐	☐	☐

Please turn over the page

SECTION 7: TESTS

	Never 1	2	3	4	5	6	Always 7	Don't know	Not Applicable
17. Do you understand the **reasons** for your tests?	☐	☐	☐	☐	☐	☐	☐	☐	☐
18. Do you get your test results back within an acceptable time period?	☐	☐	☐	☐	☐	☐	☐	☐	☐
19. Do you understand the **results** of your tests?	☐	☐	☐	☐	☐	☐	☐	☐	☐

SECTION 8: SHARING DECISIONS ABOUT YOUR CARE

Does the renal team:

	Never 1	2	3	4	5	6	Always 7	Don't know	Not Applicable
20. Talk with you about your treatment and life goals?	☐	☐	☐	☐	☐	☐	☐	☐	☐
21. Enable you to participate in decisions about your kidney care as much as you want?	☐	☐	☐	☐	☐	☐	☐	☐	☐
22. Talk to you about taking a more active role in managing your own kidney care?	☐	☐	☐	☐	☐	☐	☐	☐	☐

SECTION 9: PRIVACY AND DIGNITY

	Never 1	2	3	4	5	6	Always 7	Don't know	Not Applicable
23. Are you given enough privacy when discussing your condition or treatment?	☐	☐	☐	☐	☐	☐	☐	☐	☐
24. Is your dignity respected during visits and clinical examinations?	☐	☐	☐	☐	☐	☐	☐	☐	☐

Please turn over the page

Hawkins J, *et al. BMJ Open* 2024; 11:e044984. doi: 10.1136/bmjopen-2020-044984

SECTION 10: SCHEDULING AND PLANNING

	**Never** 1	2	3	4	5	6	**Always** 7	Don't know	Not Applicable
25. Can you change your appointment times if they are not suitable for you?	☐	☐	☐	☐	☐	☐	☐	☐	☐
26. Do you feel your time is used well at your appointments relating to your kidneys?	☐	☐	☐	☐	☐	☐	☐	☐	☐

If you are on in-hospital or in-satellite haemodialysis, please move on to SECTION 11: HOW THE RENAL TEAM TREATS YOU. If you have blood tests done at an outpatient clinic or GP surgery, please answer question 27.

	**Never** 1	2	3	4	5	6	**Always** 7	Don't know	Not Applicable
27. Are the arrangements for your blood tests convenient for you?	☐	☐	☐	☐	☐	☐	☐	☐	☐

SECTION 11: HOW THE RENAL TEAM TREATS YOU

Thinking about how the renal team treats you, do they:

	**Never** 1	2	3	4	5	6	**Always** 7	Don't know	Not Applicable
28. Take you seriously?	☐	☐	☐	☐	☐	☐	☐	☐	☐
29. Show a caring attitude towards you?	☐	☐	☐	☐	☐	☐	☐	☐	☐
30. Ask you about your emotional feelings?	☐	☐	☐	☐	☐	☐	☐	☐	☐

Hawkins J, *et al. BMJ Open* 2024; 11:e044984. doi: 10.1136/bmjopen-2020-044984

SECTION 12: TRANSPORT

If the renal unit arranges your transport, please answer these questions.
If the unit does not arrange your transport then please move on to SECTION 13: THE ENVIRONMENT.

	Never 1	2	3	4	5	6	Always 7	Don't know	Not Applicable
31. Is the vehicle provided suitable for you?	☐	☐	☐	☐	☐	☐	☐	☐	☐
32. Is the time it takes to travel between your home and the renal unit acceptable to you?	☐	☐	☐	☐	☐	☐	☐	☐	☐
33. Once your visit to the renal unit is finished and you are ready to leave, are you able to leave within less than 30 minutes?	☐	☐	☐	☐	☐	☐	☐	☐	☐

SECTION 13: THE ENVIRONMENT

When you attend the renal unit, how would you grade:

	Poor 1	2	3	4	5	6	Excellent 7	Don't know	Not Applicable
34. Accessibility (e.g., lifts, ramps, automatic doors)?	☐	☐	☐	☐	☐	☐	☐	☐	☐
35. Comfort?	☐	☐	☐	☐	☐	☐	☐	☐	☐
36. Cleanliness?	☐	☐	☐	☐	☐	☐	☐	☐	☐
37. Waiting area?	☐	☐	☐	☐	☐	☐	☐	☐	☐
38. Parking?	☐	☐	☐	☐	☐	☐	☐	☐	☐

Please turn over the page

Hawkins J, *et al. BMJ Open* 2024; 11:e044984. doi: 10.1136/bmjopen-2020-044984

SECTION 14: YOUR OVERALL EXPERIENCE

	Worst it can be 1	2	3	4	5	6	Best it can be 7
39. How well would you grade your overall experience of the service provided by your renal unit on a scale from 1 (worst it can be) to 7 (best it can be)?	☐	☐	☐	☐	☐	☐	☐

Thank you for completing this questionnaire.

For further information please visit www.renalreg.org/projects/prem

Page 8 – PREM 2018
