# Supplementary material for: Contributions of treatment centre and patient characteristics to patient-reported experience of haemodialysis: a national cross-sectional study
Source: BMJ Open. 2021 Apr 14;11(4):e044984. doi: 10.1136/bmjopen-2020-044984 (PMC8054084; doi:10.1136/bmjopen-2020-044984)
Supplement: Author's manuscript [file bmjopen-2020-044984.draft_revisions.pdf]

BMJ Open is committed to open peer review. As part of this commitment we make the peer review history of every article we publish publicly available.

When an article is published we post the peer reviewers' comments and the authors' responses online. We also post the versions of the paper that were used during peer review. These are the versions that the peer review comments apply to.

The versions of the paper that follow are the versions that were submitted during the peer review process. They are not the versions of record or the final published versions. They should not be cited or distributed as the published version of this manuscript.

BMJ Open is an open access journal and the full, final, typeset and author-corrected version of record of the manuscript is available on our site with no access controls, subscription charges or pay-per-view fees (http://bmjopen.bmj.com).

If you have any questions on BMJ Open's open peer review process please email info.bmjopen@bmj.com

BMJ Open

Treatment centre is the main determinant of patient-reported experience of haemodialysis: a national cross-sectional study

Journal:	*BMJ Open*
Manuscript ID	bmjopen-2020-044984
Article Type:	Original research
Date Submitted by the Author:	21-Sep-2020
Complete List of Authors:	Hawkins, Janine; University of Hertfordshire, Life and Medical Sciences<br>Smeeton, Nigel; University of Hertfordshire, Health and Social Work<br>Busby, Amanda; University of Hertfordshire School of Life and Medical Sciences, Life and Medical Sciences<br>Wellsted, David; University of Hertfordshire, Health Research Methods Unit<br>Rider, Beth; University of Hertfordshire, Life and Medical Sciences<br>Jones, Julia; University of Hertfordshire, Health and Social Work<br>Steenkamp, Retha; Renal Association<br>Stannard, Catherine; Renal Association<br>Gair, Rachel; Renal Association<br>van der Veer, Sabine N.; The University of Manchester<br>Corps, Claire; Leeds Teaching Hospitals NHS Trust<br>Farrington, Ken; Lister Hospital, Renal Unit; University of Hertfordshire, Life and Medical Sciences
Keywords:	NEPHROLOGY, GENERAL MEDICINE (see Internal Medicine), Quality in health care < HEALTH SERVICES ADMINISTRATION & MANAGEMENT

SCHOLARONE™
Manuscripts

I, the Submitting Author has the right to grant and does grant on behalf of all authors of the Work (as defined in the below author licence), an exclusive licence and/or a non-exclusive licence for contributions from authors who are: i) UK Crown employees; ii) where BMJ has agreed a CC-BY licence shall apply, and/or iii) in accordance with the terms applicable for US Federal Government officers or employees acting as part of their official duties; on a worldwide, perpetual, irrevocable, royalty-free basis to BMJ Publishing Group Ltd ("BMJ") its licensees and where the relevant Journal is co-owned by BMJ to the co-owners of the Journal, to publish the Work in this journal and any other BMJ products and to exploit all rights, as set out in our licence.

The Submitting Author accepts and understands that any supply made under these terms is made by BMJ to the Submitting Author unless you are acting as an employee on behalf of your employer or a postgraduate student of an affiliated institution which is paying any applicable article publishing charge ("APC") for Open Access articles. Where the Submitting Author wishes to make the Work available on an Open Access basis (and intends to pay the relevant APC), the terms of reuse of such Open Access shall be governed by a Creative Commons licence – details of these licences and which Creative Commons licence will apply to this Work are set out in our licence referred to above.

Other than as permitted in any relevant BMJ Author's Self Archiving Policies, I confirm this Work has not been accepted for publication elsewhere, is not being considered for publication elsewhere and does not duplicate material already published. I confirm all authors consent to publication of this Work and authorise the granting of this licence.

For peer review only

Treatment centre is the main determinant of patient-reported experience of haemodialysis: a national cross-sectional study

Author list

*, **Janine Hawkins[1],*Nigel Smeeton[2],Amanda Busby[1], David Wellsted[1], Beth Rider[1],Julia Jones[2],Retha Steenkamp[3],Catherine Stannard[3],Rachel Gair[3], Sabine N van der Veer[5], Claire Corps[4], Ken Farrington[1,6]

*joint first author; **guarantor

Name	Job Title	Email
*Janine Hawkins[1]	Senior Research Fellow	j.hawkins3@herts.ac.uk
*Nigel Smeeton[2]	Senior Research Fellow/Social Statistician	n.smeeton@herts.ac.uk
Amanda Busby[1]	Senior Research Assistant	a.busby@herts.ac.uk
David Wellsted[1]	Reader in Health Research Methods	d.m.wellsted@herts.ac.uk
Beth Rider[1]	Research Assistant	b.rider@herts.ac.uk
Julia Jones[2]	Professor of Public Involvement and Health	j.jones26@herts.ac.uk
Retha Steenkamp[4]	Head of Operations	Retha.Steenkamp@renalregistry.nhs.uk
Catherine Stannard[4]	KQuIP Quality Improvement Programme Manager	Catherine.Stannard@renalregistry.nhs.uk
Rachel Gair[4]	KQuIP Programmes Lead	Rachel.Gair@renalregistry.nhs.uk
Sabine N van der Veer[5]	Lecturer in Health Data Sciences	sabine.vanderveer@manchester.ac.uk
Claire Corps[3]	Patient and Research Scientist	Claire.corps@nhs.net
Ken Farrington[1, 6]	Consultant Nephrologist and Head of Centre for Health Services and Clinical Research	Ken.farrington@nhs.net

[1]Centre for Health Services and Clinical Research, University of Hertfordshire, UK
[2]Centre for Research in Public Health and Community Care, University of Hertfordshire, UK
[3]Leeds Teaching Hospitals NHS Trust, Leeds, UK
[4]UK Renal Registry, University of Bristol, UK
[5]Centre for Health Informatics, Manchester Academic Health Science Centre, University of Manchester, UK
[6]E&N Herts NHS Trust, Stevenage, UK

Abstract 338

Body of text Words 3417

Tables 5

For peer review only - http://bmjopen.bmj.com/site/about/guidelines.xhtml

Abstract

Objectives To examine the relative importance of patient and centre level factors in determining self-reported experience of care in patients with advanced kidney disease treated by maintenance haemodialysis.

Design Analysis of data from a cross sectional national survey; the UK Renal Registry (UKRR) national chronic kidney disease (CKD) Patient Reported Experience Measure (PREM) survey (2018). Centre level data were obtained from the UK Renal Registry report (2018).

Setting National survey of patients with advanced kidney disease receiving treatment with maintenance haemodialysis in UK renal centres in 2018.

Participants The PREM was distributed to all UK renal centres by the UKRR in May 2018. Each centre invited patients receiving outpatient treatment for kidney disease to complete the PREM. These included patients with chronic kidney disease, those receiving dialysis – both haemodialysis and peritoneal dialysis, and those with functioning kidney transplant. There were no formal inclusion/exclusion criteria.

Main outcome measures The CKD PREM has 38 questions in 13 subscales. Responses were captured using a 7-point Likert scale (Never 1, Always 7). The primary outcome of interest was the mean PREM score calculated across all questions. Multilevel modelling was used to determine the proportion of variation of the mean PREM score across centres due to patient-related and centre-related factors.

Results There were records for 8253 haemodialysis patients (61% male, 77% White) from 69 renal centres (9 - 710 patients per centre). There was significant variation in mean PREM score across centres (5.35 to 6.53). In the multivariable analysis there was some variation in relation to both patient- and centre level factors but these contributed little to explaining the overall variation. However, multilevel modelling showed that the overwhelming proportion of the explained variance (45%) was explained by variation between centres (40%), only a small proportion of which is identified by measured factors. Only 5% of the variation was related to patient-level factors.

Conclusions Centre rather than patient characteristics determine the experience of care of patients receiving haemodialysis. Further work is required to define the characteristics of the treating centre which determine patient experience.

STRENGTHS AND LIMITATIONS OF THIS STUDY

- A large sample size deriving from a national study involving all but two UK renal centres
- We employ multilevel analysis to explore the factors that inform patient experience of renal care at both patient- and centre-level and provide accurate estimates of their relative contributions.
- PREM results do not take into account the potential impact of negative non-response bias, i.e. the tendency to participate in the survey being negatively associated with problem reporting.
- Whilst distribution of scores is skewed, sensitivity modelling indicated that the reported models and interpretation are robust.

For peer review only - http://bmjopen.bmj.com/site/about/guidelines.xhtml

INTRODUCTION

As the survival of patients improves, attention is shifting from clinical and biomedical “hard metrics” as primary indicators of healthcare quality (1) towards gathering feedback from patients on aspects of their experience consistent with their values; including health, wellbeing, respect and dignity, quality of life (QoL), and independence (2). Patient experience of care, defined as ‘patients’ perception of the range of interactions they have with the healthcare system they use’ (3-5) is increasingly recognised as an indicator of healthcare quality (5-7). The patient perspective has traditionally been assessed through measures of patient satisfaction (2). Patient-reported experience measures (PREMs) are emerging as a favoured metric for assessing healthcare quality (3, 7, 8). PREMs are standardised self-report tools designed to tap into specific domains of care (i.e. communication, care environment, healthcare team). Unlike satisfaction measures, patient-reported experience captures patient perspective of quality by assessing the extent to which specific care processes occur over time (3, 9) (e.g. *“In the last 12 months how often did your doctor give you the information you needed?”*). Patient experience allows providers to monitor the extent to which fundamental standards of healthcare quality have been met, through objective reporting (3, 7). In contrast, patient satisfaction measures assess the extent to which patients are *happy* with care, which is a reflection of patients’ own subjective standards, expectations and affect, which is highly influenced by patient gratitude for the care received, and provides a less reliable measure of healthcare quality (3, 7, 10). PREMs aim to minimize subjective bias that arises from patient satisfaction measures (6). Nevertheless, patient experience influences patient satisfaction, demonstrating a causal link between these quality metrics (10). Measuring patient experience offers a unique understanding of how we appraise healthcare quality, by responding to the voice of those at the centre of care (7). Evaluated alongside patient safety and effectiveness, patient experience contributes to an overall holistic representation of healthcare quality (6, 7, 11).

PREMs can be used by healthcare providers, policymakers and regulators to assess provider performance and facilitate quality improvement initiatives (1). PREMs are also used to inform pay-for-performance schemes, by which services may receive funding increments in line with their performance (8, 9). Survey results allow for comparisons across providers (1) and can be made publicly available for use by patients and the general public. Patient experience is linked to a range of outcomes across healthcare populations; including clinical effectiveness, self-rated and objectively measured health outcomes, patient activation and treatment adherence (9, 12). This demonstrates the clinical utility of measuring patient-reported experience towards optimizing healthcare delivery and patient outcomes. In the UK, approximately 6% of the adult population have a diagnosis of chronic kidney disease (CKD). Most have mild to moderate disease (Stages 1-3), with around 0.4% having advanced CKD (Stages 4 and 5). Renal replacement therapy (RRT) is

For peer review only - http://bmjopen.bmj.com/site/about/guidelines.xhtml

required for many patients with Stage 5 disease. Around 60,000 patients in the UK receive RRT, approximately half of whom are living with a renal transplant and the remainder receiving dialysis (13). Haemodialysis (HD) is the most prevalent dialysis modality. HD patients require long-term and burdensome treatment throughout their lifetime (6). The quality of this care can have a significant impact on these patients' wellbeing, health and quality of life (6, 14). Health-related quality of life (HRQoL) is often indicated by patients as being more important to them than survival (15).

Introduced in 2016, the UK Renal Registry (UKRR) CKD-PREM is part of a national program that collects annual feedback from patients with kidney disease on their experience of NHS renal services, including those on HD. However, the implementation of PREMs in clinical practice has been relatively limited (6). As a result, responding to patient experience feedback remains a challenge for providers (16). To translate patient feedback into actionable information to drive quality improvements (6), we must be able to define which factors inform patient experience. Whether patient experience is mainly determined by patient characteristics (17) or by how services respond to patients remains unclear. There is a growing body of literature addressing patient experience of care (and the factors which inform it), but few studies acknowledge the hierarchical framework of patients nested within centres. No previous studies have taken account of this framework in the haemodialysis population. The objective of the current study was to examine the relative importance of patient and centre level factors in determining self-reported experience of care in patients with advanced kidney disease treated by maintenance haemodialysis. We used clustered analysis (multilevel regression models) to examine the association between patient demographic and centre-level factors and patient experience, using secondary data from the national 2018 CKD-PREM survey. We have focused on patients receiving HD to reduce the heterogeneity of the sample and because data suggest poorer experience of care in this group (18). By using analytical methods that account for grouping within treatment centre, our study aims to provide evidence that treating centre makes a larger contribution to patient experience than patient characteristics, thereby supporting a systems perspective towards patient experience (1).

For peer review only

For peer review only - http://bmjopen.bmj.com/site/about/guidelines.xhtml

METHODS

PREM data

The validated PREM questionnaire was developed by a group of patients, clinicians and academics as a disease specific measure of patient experience. The third survey, conducted in 2018 (19), involved 71 adult renal centres across the UK, with kidney patients given the option of a paper version of the questionnaire, distributed and collected by centres, or completion online. Patient age, gender and ethnicity were recorded along with current treatment (peritoneal dialysis; haemodialysis and its location (at home, in hospital, in satellite); transplantation; or attending kidney clinics but not on dialysis or transplantation). Patients reported their use of hospital transport by answering transport-specific questions. Information regarding patient experience of care was collected from 38 questions grouped into 13 subscales consisting of between two and five questions, with a final question asking the patient to grade their overall experience of the service provided. Responses were captured using a 7-point Likert scale from Never (1) to Always (7), with additional options of Don't know and Not Applicable. The research reported here investigated only those patients receiving haemodialysis. The sample size was determined by the number of haemodialysis patients providing a completed PREM questionnaire in the 2018 survey.

Centre-level population characteristics

In addition to the patient-level survey data, the UK Renal Registry (UKRR) provides centre-level population characteristics (centre-level data). UKRR has collected centre-based data and published annual reports since 1998. Information provided for this study was from 2017 (13), the most recently available. Centre demographic variables included mean patient age, percentage male, ethnicity distribution (White, Asian, Black, Other) and mean index of multiple deprivation. Follow-up information was obtained on the percentage of patients alive at the end of 2016 that died during 2017. Other centre-level information related to dialysis access for patients receiving renal replacement therapy, current or previous smoking, comorbidities at the start of renal replacement therapy (e.g. angina, previous myocardial infarction, diabetes), the percentage of patients who were late presenters (i.e. less than 90 days between first nephrology appointment and commencing RRT), and mean systolic and diastolic blood pressures. Mean blood test levels were provided for phosphate, bicarbonate, potassium, ferritin and urea reduction ratio. Information on transplant waiting lists and transplant rate was also obtained.

Analysis

The primary outcome of interest was the mean PREM score calculated across all 38 questions across the 13 subscales, analysed as a continuous variable, with a difference of greater than 10% (0.7) of the scale range (1 to 7) being regarded as clinically important. Where data were incomplete, scores were calculated if four

For peer review only - http://bmjopen.bmj.com/site/about/guidelines.xhtml

or fewer answers were missing out of the 33 questions with no filtering. Details of how the mean score was adjusted for missing responses is provided in the 2018 report (19).

The information available from the completed PREM questionnaires was used to obtain the patient-level variables, these being: age in years (17-21, 22-30, 31-40, 41-55, 56-64, 65-74, 75-84, 85+), gender (male, female, rather not say), ethnicity (White, Asian, Black, Other, rather not say), use of hospital transport (yes, no) and haemodialysis location (in-hospital, in-satellite, at home). Clinically relevant centre-level variables were selected. As the range of possible values differs between the constituent countries of the UK, for comparability, deciles for the Index of Multiple Deprivation were calculated.

A multivariable linear regression, which assumes independence of observations, was performed on the patient-level and centre-level variables that were at least 85% complete and available for each centre. Variables with a *P*-value of less than 0.1 were selected for multilevel modelling. Multilevel analyses were then performed on the mean scores by using the iterative generalised least squares method of estimation. Variance partition coefficients were calculated. These represent the proportion of total variation in an outcome that is due to differences occurring at each level; a high coefficient at centre level indicates that more of the variation in the model is due to differences between centres than between patients. Firstly, the variance partition coefficients were calculated for the null model to give raw coefficients. Then a model was fitted adjusting for patient-level characteristics to give patient adjusted coefficients. Next, both patient-level and centre-level characteristics were entered. Finally, any interactions between variables identified were added in to give fully adjusted coefficients.

Sets of models were compared using likelihood ratio tests. In order to assess whether the patients entered into the analysis were similar to those excluded due to incomplete information, the two groups were compared on age group, gender, ethnicity and location of treatment.

Sensitivity analyses were performed as part of the modelling. As the PREM scores were negatively skewed, an exponential transformation was applied to bring the observations closer to a normal distribution. Additionally, results were compared with and without the robust estimation of errors. Snijders/Bosker R-squared values were estimated after fitting the multilevel model to determine the proportion of variance in scores attributable to centre-level and patient-level differences.

All analyses were conducted using Stata version IC/15.1.

Patient and Public Involvement

The UKRR Patient Council was consulted regarding how best to share this research, as well as the Kidney PREM and other related research, with patients, medical professionals and the public in general. The study

For peer review only - http://bmjopen.bmj.com/site/about/guidelines.xhtml

For peer review only

was presented at a Patient Council meeting in March 2019 and the members' views sought in particular on our dissemination plans to maximise the impact of the research on patient care.

Feedback from the Council was that there are many ways to engage with the Kidney community such as through public user group forums, patient blogs or via the Kidney Patient Associations (KPA). It was also felt that it would be beneficial to send results directly to unit staff, as well as the hospital chief executive, medical director and clinical director to ensure the findings were received at all organisational levels. In addition, the Council were keen that units were sent their own results from the PREM, highlighting how they compare to other units, to ensure that best practice was shared across settings.

For peer review only

For peer review only - http://bmjopen.bmj.com/site/about/guidelines.xhtml

RESULTS

There were records for 8253 haemodialysis patients from 69 renal centres (9 to 710 patients per centre) with two centres providing no responses. Overall, reported experience of PREM was positive (mean = 5.93, standard deviation = 1.00). There was noticeable variation between centres; with mean scores ranging from 5.35 to 6.53. This range (1.18) is far greater than 10% (0.7) of the scale range (1 to 7) and more than 1 standard deviation of the overall score, giving an indication of the impact of the centre-level variation. Table 1 shows PREM summary scores for groups based on demographic characteristics. Of note, those unwilling to disclose their gender or ethnicity (selecting "Rather Not Say") on average gave lower experience scores. Some variation was seen across age groups, with those over 65 years reporting a better experience. However, the difference was small, ranging from 5.71 (18-21 years) to 6.01 (85+ years).

For peer review only

For peer review only - http://bmjopen.bmj.com/site/about/guidelines.xhtml

Table 1. Mean reported patient experience scores by patient characteristics in the analysed sample (percentages out of patients with the information present, *n* = 8253)

Variable	Observations	Mean	Standard deviation	Median
Age (years)				
17-21	28 (0.3%)	5.71	1.04	5.94
22-30	160 (2.0%)	5.79	1.07	6.05
31-40	325 (4.0%)	5.87	1.10	6.28
41-55	1314 (16.1%)	5.80	1.11	6.14
56-64	1540 (18.9%)	5.86	1.06	6.15
65-74	2255 (27.7%)	6.00	0.96	6.31
75-84	2046 (25.1%)	6.01	0.91	6.27
85+	477 (5.9%)	6.01	0.94	6.28
Missing	108			
Gender				
Male	4481 (60.8%)	5.96	0.98	6.26
Female	2861 (38.8%)	5.91	1.00	6.21
Not say	33 (0.4%)	4.43	1.42	4.36
Missing	878			
Ethnicity				
White	6025 (77.3%)	5.98	0.97	6.28
Asian	893 (11.5%)	5.74	1.05	5.97
Black	541 (6.9%)	5.83	1.04	6.09
Other	210 (2.7%)	5.99	0.93	6.27
Not say	129 (1.7%)	5.12	1.40	5.29
Missing	455			
Location of treatment				
Home	270 (3.4%)	6.12	0.91	6.42
Satellite	4131 (51.6%)	5.93	1.00	6.24
Hospital	3600 (45.0%)	5.93	1.01	6.22
Missing	252			
Hospital transport				
Yes	5226 (63.3%)	5.90	1.01	6.20
No	3027 (36.7%)	5.98	0.99	6.29

For peer review only

For peer review only - http://bmjopen.bmj.com/site/about/guidelines.xhtml

Missing	0

Those centre-level variables with at least 85% completeness and recorded by all renal centres are shown in Table 2. These consisted of socio-demographic variables at both patient summary and centre levels, centre size, transplanting centre (yes/no), blood test results and waiting list information. Although the data for percentage White at renal centre level were more than 85% complete, they were unavailable for 7 centres, all in Scotland, and so were excluded. However, there was a strong negative correlation between percentage White and size of centre (r = –0.7922) suggesting that centre size is a good proxy variable for percentage White.

Table 2. Descriptive statistics for centres

	N	Mean	Standard deviation	Range Across Centres
Mean Age* (years)	69	65.38	2.46	59.96 - 72.76
Percentage Male*	69	61.98	4.06	47.14 - 70.27
Mean Index of Multiple Deprivation Decile*	69	4.74	1.13	1 - 7
Transplanting Centre*				
Yes	22 (31.9%)			
No	47 (68.1%)			
Size of Centre*	69	891.01	684.68	124 – 3417
Mean Haemoglobin Level (g/L)*	69	109.20	2.59	103.4 - 115.98
Mean Phosphate Level (mmol/L)	69	1.54	0.13	0.79 - 1.74
Mean Ferritin (ng/mL)	66	491.71	115.82	297.10 - 888.79
Wait Listed within 2 years starting RRT (%)*	69	40.87	11.61	14.29 - 65.38
Transplant within 2 years of waiting list (%)	69	53.43	15.30	25 – 100
% Died with 90 days starting RRT	69	16.55	3.34	10.18 - 25.69

The results from the multivariable linear regression are shown in Table 3. All patient level variables were retained in the model, along with selected centre-level variables as indicated in Table 2. Adjusting for other patient and centre-level variables increased the effect of age on the PREM scores, with a wider range of 0.53 across the age groups (compared to 0.3 as shown in Table 1). Several centre-level variables were found to be reliably associated with mean scores in the adjusted model, however the range of effect for any variable was limited (95% confidence interval range between .03, and .44).

For peer review only - http://bmjopen.bmj.com/site/about/guidelines.xhtml

Table 3. Results for the multivariable linear regression. (*n* = 6795, 69 centres)

Variable	Coefficient	95% confidence interval	P-value
**Patient-level**			
Age (baseline 56-64)			
17-21	-0.334	(-0.734, 0.067)	0.102
22-30	-0.058	(-0.233, 0.117)	0.515
31-40	0.028	(-0.101, 0.156)	0.673
41-55	-0.032	(-0.111, 0.047)	0.424
56-64	-	-	-
65-74	0.119	(0.050, 0.187)	0.001
75-84	0.146	(0.075, 0.217)	<0.001
85+	0.197	(0.087, 0.307)	<0.001
Gender (baseline male)			
Female	-0.030	(-0.078, 0.018)	0.223
Not say	-1.072	(-1.468, -0.677)	<0.001
Ethnicity (baseline White)			
Asian	-0.054	(-0.135, 0.027)	0.193
Black	-0.078	(-0.176, 0.020)	0.121
Other	0.125	(-0.019, 0.270)	0.089
Not say	-0.627	(-0.826, -0.429)	<0.001
Location of treatment (baseline home)			
Satellite	-0.149	(-0.282, -0.016)	0.028
Hospital	-0.219	(-0.353, -0.086)	0.001
Hospital transport	-0.120	(-0.170, -0.070)	<0.001
**Centre-level**			
Mean age (per year)	0.024	(0.009, 0.039)	0.002
Percentage male (per 10%)	-0.110	(-0.185, -0.036)	0.004
Mean IMD decile	-0.057	(-0.086, -0.027)	<0.001
Transplant centre	0.274	(0.200, 0.348)	<0.001
Centre size (per 100 patients)	-0.024	(-0.030, -0.018)	<0.001
Mean haemoglobin (g/L)	0.013	(0.002 0.024)	0.026
Mean phosphate (mmol/L)	0.022	(-0.197, 0.240)	0.846
Mean ferritin (ng/mL)	-0.0001	(-0.0003, 0.0001)	0.297
On waiting list < 2 years from starting RRT (per 10%)	0.075	(0.049, 0.102)	<0.001
Transplant within 2 years of waiting list (per 10%)	0.015	(-0.007, 0.037)	0.184
% dead within 90 days of starting RRT (per 10%)	-0.016	(-0.108, 0.076)	0.736

Table 4 provides details of relationships between the explanatory variables at the level of centre and patient, and the mean patient experience score. Data for the multilevel modelling were obtained from

For peer review only - http://bmjopen.bmj.com/site/about/guidelines.xhtml

6945 (84.2%) patients, with 69 centres represented. Patients who were included differed from those excluded in terms of ethnicity (White: 77.8% v. 72.6%) and location of treatment (hospital: 45.4% v. 42.0%). However, age (65+: 58.8% v. 57.9%), gender (male: 60.8% v. 59.5%) and use of hospital transport (yes: 62.9% v. 65.8%) were similar.

Table 4. Results for the multilevel modelling. (*n* = 6945, 69 centres)

Variable	Coefficient	95% confidence interval	P-value
**Patient-level**			
Age (baseline 56-64)			
17-21	-0.359	(-0.806, 0.089)	0.116
22-30	-0.088	(-0.287, 0.112)	0.389
31-40	0.022	(-0.098, 0.142)	0.715
41-55	-0.035	(-0.116, 0.046)	0.396
65-74	0.118	(0.047, 0.188)	0.001
75-84	0.147	(0.083, 0.212)	<0.001
85+	0.219	(0.124, 0.313)	<0.001
Gender (baseline male)			
Female	-0.027	(-0.076, 0.022)	0.275
Not say	-1.074	(-1.662, -0.485)	<0.001
Ethnicity (baseline White)			
Asian	-0.054	(-0.166, 0.057)	0.339
Black	-0.052	(-0.162, 0.058)	0.352
Other	0.122	(-0.059, 0.304)	0.186
Not say	-0.625	(-0.916, -0.334)	<0.001
Location of treatment (baseline home)			
Satellite	-0.127	(-0.258, 0.005)	0.060
Hospital	-0.231	(-0.383, -0.079)	0.003
Hospital transport	-0.128	(-0.183, -0.072)	<0.001
**Centre-level**			
Mean age (per year)	0.015	(-0.012, 0.043)	0.268
Percentage male (per 10%)	-0.122	(-0.232, -0.011)	0.031
Mean IMD decile	-0.035	(-0.086, 0.016)	0.174
Transplant centre	0.291	(0.165, 0.416)	<0.001
Centre size (per 100 patients)	-0.027	(-0.037, -0.018)	<0.001
Mean haemoglobin (g/L)	0.011	(-0.009, 0.031)	0.267
On waiting list < 2 years from starting RRT (per 10%)	0.055	(0.015, 0.095)	0.007

At the centre level, higher mean scores were associated with a higher percentage placed on the waiting list for a transplant within two years of starting renal replacement therapy. Lower scores were associated with larger centres. At the patient level, lower mean scores were associated with younger patients (< 56 years),

For peer review only - http://bmjopen.bmj.com/site/about/guidelines.xhtml

refusal to disclose gender, refusal to disclose ethnicity, use of hospital transport and receipt of HD in-centre or in-satellite (compared with dialysis at home). Most of these effects were relatively small (< 0.4 on a scale from 1 to 7), the main exceptions being refusal to disclose gender and refusal to disclose ethnicity. Patient-level variables had a similar effect on scores in the multilevel model as they did in the multivariable linear regression model (tables 3 and 4). The sensitivity analyses performed by firstly dropping robust estimation and then using the transformed data each produced similar results to those of the main analysis.

Table 5 shows the estimated variance for each model. The total variance decreases as the patient-level variables are added and decreases further once adjusted for centre-level variables (i.e. as more of the variance in patient scores was explained). The Snijders-Bosker R-squared values (20) suggest that 40.1% of variation in scores is explained by centre variation and just 5.3% of variation is accounted for by differences in patient characteristics.

Table 5: Variance at level of centre and patient, before and after adjustment for explanatory variables

Level of adjustment	Variance (total)	Variance (cons)	Variance (residual)	Variance accounted for	
				Patient level variables (%)*	Centre level variables (%)*
Unadjusted	1.0166	0.0407	0.9759		
Adjusted for patient level variables	0.9763	0.0433	0.9330		
Adjusted for patient and centre level variables	0.9486	0.0187	0.9299	5.3	40.1

For peer review only - http://bmjopen.bmj.com/site/about/guidelines.xhtml

DISCUSSION

Statement of principal findings

This study provides important insights into what influences patient experience of care in HD patients in this national sample of respondents of the UKRR CKD-PREM (2018 survey). Patient characteristics - younger patients (< 56 years), refusal to disclose gender, refusal to disclose ethnicity, use of hospital transport and receipt of treatment in centre or satellite - were only modestly associated with patient experience. These effects are small in magnitude, indicating a limited impact on patient experience. Collectively, patient characteristics account for just 5% of the observed variance in the PREM score. The only important effects were observed consistently in those patients who refused to disclose their gender or ethnicity.

In contrast, the centre accounts for an overwhelming proportion of the variance (40%). However, our findings indicate that while centre plays a major role in determining patient experience of care, the centre-level factors that drive patient experience are unknown. Any associations between collected centre factors and patient experience were modest and of doubtful clinical importance (i.e. <10% (0.7) of the 1 to 7 scale range) and likely to have limited impact on patient experience. For example, lower scores were associated with larger centres (-0.027 per 100 patients, p<0.001) and higher scores associated with a higher percentage of patients placed on the transplant waiting list within two years of starting RRT (0.055 per 10%, p=0.007). These findings challenge the concept that providing care focused on patient characteristics is the key determinant of patient experience.

Factors at a group (cluster) level (for example, hospitals or GP practices) can have an influence on an individual's outcome of interest. This is typically handled in analysis using a group variable, or multi-level models. In sample size estimation where no current estimate of the "cluster effect" is published it is generally accepted that a reasonable conservative estimate of the intra-cluster correlation (ICC) is in the order of r=0.05 (21). Often, the effect of clustering is smaller. In this paper however we present evidence that indicates that for patient experience the variation between clusters (e.g. treating centres, i.e. the centre effect) is larger by several orders of magnitude. In fact, variation attributable to the observed patient-level variables (age, ethnicity and so on) is approximately 5%, whilst the variation attributable to the centre effect is approximately 40%.

Strengths and limitations of the study

Our study has considerable strengths, most notably the large sample size deriving from a national study involving all but two of the UK renal centres making it more likely that the sample was representative, raising statistical power and hence the strength of the findings. The study is also the first to employ multilevel analysis to explore the factors that inform patient experience of renal care at both patient- and

For peer review only

centre-level and provide accurate estimates of the relative contributions for factors at both these levels. It has been suggested that centre-level numbers in excess of 50 are required to prevent bias in estimates of centre-level standard errors (22). It is worth noting that the very large sample size means that a number of so-called statistically significant effects (p values <0.05) represent small differences unlikely to be of clinical importance.

The study also has some limitations. Though PREM scores were generally high, the results do not take into account the potential impact of negative non-response bias, i.e. the tendency to participate in the survey being negatively associated with problem reporting (23). Furthermore, the distribution of scores is skewed, but sensitivity modelling indicated that the reported models and interpretation are robust. More importantly, the observed variation between centres was largely unmeasured. While there have been some calls to focus on variation between centres, there is as yet little effort to understand what these factors are. Based on our study findings, the need to address this gap is urgent.

Meaning of the study: possible explanations and implications for clinicians and policymakers

These findings have implications for care delivery, suggesting that the benefits on patient experience of personalising care delivery according to patient characteristics may be far outweighed by efforts focused on reducing variation between centres as suggested by others (1, 24). However, it is unclear just what the centre characteristics are which drive this variation. Certainly, the factors examined in this study do not provide an adequate explanation. Wider systemic problems such as understaffing, lack of managerial support, and increased pressure to meet ambitious national targets can promote a culture of care delivery which is task- rather than patient-focused (25). Though these issues reflect challenges faced universally by the NHS, the centre-wide variation in patient experience observed in this study may indicate that some centres are better equipped than others to cope with increasing pressures, while others may lack the culture and mechanism to deliver the same standard of care (1).

Unanswered questions and future research

Further study is needed to determine those centre characteristics which most influence the experience of care reported by the UK haemodialysis population. The recognition of the need for 'deep cultural change' (26) in promoting quality improvement is consistent with these interpretations. Additionally, further study is needed to ascertain if this is the case across other service provisions, not just in renal settings.

In summary, this large national study of patients receiving haemodialysis suggests that it is centre rather than patient characteristics which are important in shaping patient experience of care. Our findings

For peer review only - http://bmjopen.bmj.com/site/about/guidelines.xhtml

suggest that a shift in focus from patient characteristics to the service itself is required to understand and improve patient experience of haemodialysis and potentially of many other areas of healthcare. This study provides a clear direction for future research aimed at determining the nature of the centre characteristics which drive patient experience.

For peer review only

For peer review only - http://bmjopen.bmj.com/site/about/guidelines.xhtml

WHAT IS ALREADY KNOWN ON THIS TOPIC?

Haemodialysis is necessary to maintain life in many patients with advanced kidney disease but is demanding and can be burdensome.

Both patient and centre factors influence how patients experience care.

The relative importance of these factors and the mechanisms underlying their associations with patient experience are poorly understood.

WHAT THIS STUDY ADDS

Treatment centre is the dominating factor determining patient experience with only a small amount of the reported variation being explained by patient characteristics.

Research is required to focus on defining those characteristics of the treating centre which drive patient experience.

Acknowledgements

We are grateful to the UK Renal Registry for supplying the data from the 2018 PREM and 2017 UK Renal Registry Report for analysis for this study, and to the UK Renal Registry Patient Council and for Kidney Care UK for their input. We also thank all the UK renal centres for providing data to the UK Renal Registry. Thanks too to all the patients who completed the 2018 PREM

Ethics Approval

The study involved secondary analysis of robustly anonymised UK Renal Registry data. The UK Renal Registry has NHS Health Research Authority research ethics approval (16/NE/0042).

Funding

This project was funded internally by the University of Hertfordshire with a cross-school collaboration research grant.

Author Contributions

Study design: KF, JH, JJ, NS, DW.

PPI: CC, JH, JJ.

Aggregation of data: AB, JH, NS, RS.

For peer review only - http://bmjopen.bmj.com/site/about/guidelines.xhtml

Analysis of data: AB, KF, NS, SNvdV, DW.

Interpretation of data/results: AB, CC, KF, RG, JH, JJ, BR, CS, NS, SNvdV, DW.

Drafting manuscript: AB, KF, JH, JJ, BR, NS, SNvdV, DW

Approval of final Manuscript: All

Competing Interests

All authors have completed the ICMJE uniform disclosure form at www.icmje.org/coi_disclosure.pdf and declare: no support from any organisation for the submitted work; no financial relationships with any organisations that might have an interest in the submitted work in the previous three years; no other relationships or activities that could appear to have influenced the submitted work.

Transparency Statement

The lead authors affirm that the manuscript is an honest, accurate, and transparent account of the study being reported; that no important aspects of the study have been omitted; and that any discrepancies from the study as originally planned have been explained.

Data Availability

Data are held by the UK Renal Registry. To apply to access Registry data see https://www.renalreg.org/about-us/working-with-us/

Copyright

The Corresponding Author has the right to grant on behalf of all authors and does grant on behalf of all authors, an exclusive licence (or non exclusive for government employees) on a worldwide basis to the BMJ Publishing Group Ltd to permit this article (if accepted) to be published in BMJ editions and any other BMJPGL products and sublicences such use and exploit all subsidiary rights, as set out in our licence.

For peer review only

REFERENCES

1. Raleigh VS, Frosini F, Sizmur S, Graham C. Do some trusts deliver a consistently better experience for patients? An analysis of patient experience across acute care surveys in English NHS trusts. BMJ quality & safety. 2012;21(5):381-90.
2. LaVela SL, & Gallan, A. . Evaluation and measurement of patient experience. . Patient Experience Journal. 2014;1(1):28-36.
3. Cavanaugh KL. Patient Experience Assessment is a Requisite for Quality Evaluation: A Discussion of the In-Center Hemodialysis Consumer Assessment of Health Care Providers and Systems (ICH CAHPS) Survey. Seminars in dialysis. 2016;29(2):135-43.
4. Richardson MM, Paine SS, Grobert ME, Stidley CA, Gabbay E, Harford AM, et al. Satisfaction with Care of Patients on Hemodialysis. Clinical journal of the American Society of Nephrology : CJASN. 2015;10(8):1428-34.
5. Rhee CM, Brunelli SM, Subramanian L, Tentori F. Measuring patient experience in dialysis: a new paradigm of quality assessment. Journal of nephrology. 2018;31(2):231-40.
6. Aiyegbusi OL, Kyte D, Cockwell P, Anderson N, Calvert M. A patient-centred approach to measuring quality in kidney care: patient-reported outcome measures and patient-reported experience measures. Current opinion in nephrology and hypertension. 2017;26(6):442-9.
7. Black N, Jenkinson C. Measuring patients' experiences and outcomes. BMJ (Clinical research ed). 2009;339:b2495.
8. Salisbury C, Wallace M, Montgomery AA. Patients' experience and satisfaction in primary care: secondary analysis using multilevel modelling. BMJ (Clinical research ed). 2010;341:c5004.
9. Anhang Price R, Elliott MN, Zaslavsky AM, Hays RD, Lehrman WG, Rybowski L, et al. Examining the role of patient experience surveys in measuring health care quality. Medical care research and review : MCRR. 2014;71(5):522-54.
10. Kupfer JM, Bond EU. Patient satisfaction and patient-centered care: necessary but not equal. Jama. 2012;308(2):139-40.
11. Darzi L, & Johnson, A. . High quality care for all: NHS next stage review final report.: The Stationery Office.; 2008.
12. Doyle C, Lennox L, Bell D. A systematic review of evidence on the links between patient experience and clinical safety and effectiveness. BMJ open. 2013;3(1).
13. UK Renal Registry. 21st Annual Report. 2018.
14. Palmer SC, de Berardis G, Craig JC, Tong A, Tonelli M, Pellegrini F, et al. Patient satisfaction with in-centre haemodialysis care: an international survey. BMJ open. 2014;4(5):e005020.
15. Moss AH, Davison SN. How the ESRD quality incentive program could potentially improve quality of life for patients on dialysis. Clinical journal of the American Society of Nephrology : CJASN. 2015;10(5):888-93.
16. De Silva D. Measuring patient experience. The Health Foundation.; 2013.
17. van der Veer SN, Arah OA, Visserman E, Bart HA, de Keizer NF, Abu-Hanna A, et al. Exploring the relationships between patient characteristics and their dialysis care experience. Nephrology, dialysis, transplantation : official publication of the European Dialysis and Transplant Association - European Renal Association. 2012;27(11):4188-96.
18. Song MK, Gilet CA, Lin FC, MacHardy N, DeVitoDabbs AJ, Fine JP, et al. Characterizing daily life experience of patients on maintenance dialysis. Nephrology, dialysis, transplantation : official publication of the European Dialysis and Transplant Association - European Renal Association. 2011;26(11):3671-7.
19. UK Renal Registry and Kidney Care UK. Patient Reported Experience of Kidney Care in the UK. 2018 [Available from: https://www.renalreg.org/wp-content/uploads/2017/05/PREM-report-2018-final.pdf
20. Snijders TAB, & Bosker, R. J. . Multilevel analysis: an introduction to basic and advanced multilevel modelling. . London: Sage; 2012.
21. van Breukelen GJ, Candel MJ. Calculating sample sizes for cluster randomized trials: we can keep it simple and efficient! Journal of clinical epidemiology. 2012;65(11):1212-8.
22. Maas CJM, Hos JJ. Sufficient Sample Size for Multilevel Modelling. Methodology. 2005;1 (3):86 -92.
23. Perneger TV, Chamot E, Bovier PA. Nonresponse bias in a survey of patient perceptions of hospital care. Medical care. 2005;43(4):374-80.

For peer review only - http://bmjopen.bmj.com/site/about/guidelines.xhtml

24. Senn S. Mastering variation: variance components and personalised medicine. Statistics in medicine. 2016;35(7):966-77.

25. Dixon-Woods M, Baker R, Charles K, Dawson J, Jerzembek G, Martin G, et al. Culture and behaviour in the English National Health Service: overview of lessons from a large multimethod study. BMJ quality & safety. 2014;23(2):106-15.

26. High Quality Care for All: The NHS Next Stage Review Final Report. https://assets.publishing.service.gov.uk/government/uploads/system/uploads/attachment_data/file/228836/7432.pdf. Department of Health; 2008.

For peer review only

For peer review only - http://bmjopen.bmj.com/site/about/guidelines.xhtml

The Annual Survey of Patient Reported Experience Measures (PREM)

The annual PREM survey has been designed by patients and professionals working together to find out how you feel about the services your kidney unit provides. The survey gives us feedback on renal services both locally for your unit, and nationally, and ensures that the views of kidney patients are heard. From this information, we can see what we are doing well and where we can do better. Your views matter and we act on them to help us improve services. We will provide you with feedback on the results. Information on the national results can be obtained from the UK Renal Registry or Kidney Care UK websites, along with the previous year's results.

The survey is completely anonymous, your name will not appear anywhere on the survey.

Completing the survey

The survey should take about 15 minutes to complete. Please only tick one box for each question or statement, otherwise your answer will not count.

If you prefer you can complete the survey online at www.renalreg.org/projects/prem. The online survey is available in English, Welsh, Urdu and Gujarati. Please only complete one paper PREM or one online, not both.

You can ask your partner, a friend or family member to help you complete the survey. Choosing not to take part will not affect your care in any way. When you complete the survey think about your experience of care during the last few times that you have attended. Please fill in the survey as truthfully as possible.

On completion

Please place the completed questionnaire in the envelope provided, seal it, and post it in the post box or hand it to a staff member. By completing the questionnaire you are consenting to your answers being sent to and held by the UK Renal Registry and your renal unit.

If you have any questions or concerns about the survey please contact the UK Renal Registry by emailing Catherine.stannard@renalregistry.nhs.uk or by calling 0117 414 8151.

Please complete the name of the renal/satellite unit where you are completing this survey from.

UKRR Code ☐☐☐☐☐☐☐ (To be filled in by a member of staff)

Renal Unit ☐☐☐☐☐☐☐☐☐☐☐☐☐☐☐☐☐☐☐☐

Current treatment ☐ Peritoneal dialysis ☐ Haemodialysis ☐ Transplant ☐ Attending kidney clinic but not on dialysis or transplantation

If you currently receive Haemodialysis, do you receive this…

☐ At Home ☐ In-Hospital ☐ In-Satellite

For peer review only - http://bmjopen.bmj.com/site/about/guidelines.xhtml
Please turn over the page

Age ☐ 17-21 ☐ 22-30 ☐ 31-40 ☐ 41-55

☐ 56-64 ☐ 65-74 ☐ 75-84 ☐ 85+

Gender ☐ Male ☐ Female ☐ I would rather not say

Ethnicity ☐ Asian ☐ Black ☐ White ☐ Other ☐ I would rather not say

Do you use PatientView? ☐ Yes ☐ No ☐ Don't Know

The following questions ask you about your experience with the renal unit, your kidney disease and treatment.

All the questions are answered on a scale of 1 to 7 where 1 is negative and 7 is positive.

For each question there's also a 'don't know' and 'not applicable' option.

SECTION 1: ACCESS TO THE RENAL TEAM

	Never 1	2	3	4	5	6	Always 7	Don't know	Not Applicable
1. Does the renal team take time to answer your questions about your kidney disease or treatment?	☐	☐	☐	☐	☐	☐	☐	☐	☐
2. Would you feel comfortable to contact the unit from home if you were anxious or worried?	☐	☐	☐	☐	☐	☐	☐	☐	☐
3. Would you feel able to ask for an additional appointment with your kidney doctor if you wanted to?	☐	☐	☐	☐	☐	☐	☐	☐	☐

For peer review only - http://bmjopen.bmj.com/site/about/guidelines.xhtml
Please turn over the page

SECTION 2: SUPPORT

Does the renal team help you to get the support you want with:

	Never 1	2	3	4	5	6	Always 7	Don't know	Not Applicable
4. Medical issues resulting from your kidney disease?	☐	☐	☐	☐	☐	☐	☐	☐	☐
5. Any other concerns or anxieties resulting from your kidney disease or treatment?	☐	☐	☐	☐	☐	☐	☐	☐	☐
6. Accessing patient support groups such as Kidney Patient Associations (KPA)?	☐	☐	☐	☐	☐	☐	☐	☐	☐

SECTION 3: COMMUNICATION

Do you think there is good communication between:

	Never 1	2	3	4	5	6	Always 7	Don't know	Not Applicable
7. You and your renal team?	☐	☐	☐	☐	☐	☐	☐	☐	☐
8. Members of the renal team?	☐	☐	☐	☐	☐	☐	☐	☐	☐
9. Your GP and the renal team?	☐	☐	☐	☐	☐	☐	☐	☐	☐
10. The renal team and other medical specialists?	☐	☐	☐	☐	☐	☐	☐	☐	☐
11. The renal team and other non-healthcare services if you need them, such as social work or housing?	☐	☐	☐	☐	☐	☐	☐	☐	☐

For peer review only

SECTION 4: PATIENT INFORMATION

Does the renal team:

	Never 1	2	3	4	5	6	Always 7	Don't know	Not Applicable
12. Explain things to you in a way that is easy to understand?	☐	☐	☐	☐	☐	☐	☐	☐	☐
13. Give you as much information about your kidney disease or treatment as you want?	☐	☐	☐	☐	☐	☐	☐	☐	☐

SECTION 5: FLUID INTAKE AND DIET

Thinking about the advice you are given about <u>fluid intake</u>:

	Never 1	2	3	4	5	6	Always 7	Don't know	Not Applicable
14. Does the renal team give you clear advice on your **fluid intake**?	☐	☐	☐	☐	☐	☐	☐	☐	☐

Thinking about the advice you are given about <u>diet</u>:

	Never 1	2	3	4	5	6	Always 7	Don't know	Not Applicable
15. Does the renal team give you clear advice on your **diet**?	☐	☐	☐	☐	☐	☐	☐	☐	☐

SECTION 6: NEEDLING

If you are on in-hospital or in-satellite haemodialysis please answer question 16, otherwise please go to SECTION 7: TESTS

	Never 1	2	3	4	5	6	Always 7	Don't know	Not Applicable
16. How often do the renal team insert your needles with as little pain as possible?	☐	☐	☐	☐	☐	☐	☐	☐	☐

For peer review only

SECTION 7: TESTS

	Never 1	2	3	4	5	6	Always 7	Don't know	Not Applicable
17. Do you understand the **reasons** for your tests?									
18. Do you get your test results back within an acceptable time period?									
19. Do you understand the **results** of your tests?									

SECTION 8: SHARING DECISIONS ABOUT YOUR CARE

Does the renal team:

	Never 1	2	3	4	5	6	Always 7	Don't know	Not Applicable
20. Talk with you about your treatment and life goals?									
21. Enable you to participate in decisions about your kidney care as much as you want?									
22. Talk to you about taking a more active role in managing your own kidney care?									

SECTION 9: PRIVACY AND DIGNITY

	Never 1	2	3	4	5	6	Always 7	Don't know	Not Applicable
23. Are you given enough privacy when discussing your condition or treatment?									
24. Is your dignity respected during visits and clinical examinations?									

For peer review only

For peer review only - http://bmjopen.bmj.com/site/about/guidelines.xhtml

SECTION 10: SCHEDULING AND PLANNING

	Never 1	2	3	4	5	6	Always 7	Don't know	Not Applicable
25. Can you change your appointment times if they are not suitable for you?	☐	☐	☐	☐	☐	☐	☐	☐	☐
26. Do you feel your time is used well at your appointments relating to your kidneys?	☐	☐	☐	☐	☐	☐	☐	☐	☐

If you are on in-hospital or in-satellite haemodialysis, please move on to SECTION 11: HOW THE RENAL TEAM TREATS YOU. If you have blood tests done at an outpatient clinic or GP surgery, please answer question 27.

	Never 1	2	3	4	5	6	Always 7	Don't know	Not Applicable
27. Are the arrangements for your blood tests convenient for you?	☐	☐	☐	☐	☐	☐	☐	☐	☐

SECTION 11: HOW THE RENAL TEAM TREATS YOU

Thinking about how the renal team treats you, do they:

	Never 1	2	3	4	5	6	Always 7	Don't know	Not Applicable
28. Take you seriously?	☐	☐	☐	☐	☐	☐	☐	☐	☐
29. Show a caring attitude towards you?	☐	☐	☐	☐	☐	☐	☐	☐	☐
30. Ask you about your emotional feelings?	☐	☐	☐	☐	☐	☐	☐	☐	☐

For peer review only - http://bmjopen.bmj.com/site/about/guidelines.xhtml
Please turn over the page
For peer review only

SECTION 12: TRANSPORT

If the renal unit arranges your transport, please answer these questions.
If the unit does not arrange your transport then please move on to SECTION 13: THE ENVIRONMENT.

	Never 1	2	3	4	5	6	Always 7	Don't know	Not Applicable
31. Is the vehicle provided suitable for you?	☐	☐	☐	☐	☐	☐	☐	☐	☐
32. Is the time it takes to travel between your home and the renal unit acceptable to you?	☐	☐	☐	☐	☐	☐	☐	☐	☐
33. Once your visit to the renal unit is finished and you are ready to leave, are you able to leave within less than 30 minutes?	☐	☐	☐	☐	☐	☐	☐	☐	☐

SECTION 13: THE ENVIRONMENT

When you attend the renal unit, how would you grade:

	Poor 1	2	3	4	5	6	Excellent 7	Don't know	Not Applicable
34. Accessibility (e.g., lifts, ramps, automatic doors)?	☐	☐	☐	☐	☐	☐	☐	☐	☐
35. Comfort?	☐	☐	☐	☐	☐	☐	☐	☐	☐
36. Cleanliness?	☐	☐	☐	☐	☐	☐	☐	☐	☐
37. Waiting area?	☐	☐	☐	☐	☐	☐	☐	☐	☐
38. Parking?	☐	☐	☐	☐	☐	☐	☐	☐	☐

For peer review only - http://bmjopen.bmj.com/site/about/guidelines.xhtml

Please turn over the page

SECTION 14: YOUR OVERALL EXPERIENCE

	Worst it can be 1	2	3	4	5	6	Best it can be 7
39. How well would you grade your overall experience of the service provided by your renal unit on a scale from 1 (worst it can be) to 7 (best it can be)?	☐	☐	☐	☐	☐	☐	☐

For peer review only

Thank you for completing this questionnaire.

For further information please visit www.renalreg.org/projects/prem

STROBE Statement

Checklist of items that should be included in reports of observational studies

Section/Topic	Item No	Recommendation	Reported on Page No
**Title and abstract**	1	(*a*) Indicate the study's design with a commonly used term in the title or the abstract	1
		(*b*) Provide in the abstract an informative and balanced summary of what was done and what was found	2
**Introduction**			
Background/rationale	2	Explain the scientific background and rationale for the investigation being reported	3-4
Objectives	3	State specific objectives, including any prespecified hypotheses	4
**Methods**			
Study design	4	Present key elements of study design early in the paper	5
Setting	5	Describe the setting, locations, and relevant dates, including periods of recruitment, exposure, follow-up, and data collection	5
Participants	6	(*a*) *Cohort study*—Give the eligibility criteria, and the sources and methods of selection of participants. Describe methods of follow-up<br>*Case-control study*—Give the eligibility criteria, and the sources and methods of case ascertainment and control selection. Give the rationale for the choice of cases and controls<br>*Cross-sectional study*—Give the eligibility criteria, and the sources and methods of selection of participants	5
		(*b*) *Cohort study*—For matched studies, give matching criteria and number of exposed and unexposed<br>*Case-control study*—For matched studies, give matching criteria and the number of controls per case	n/a
Variables	7	Clearly define all outcomes, exposures, predictors, potential confounders, and effect modifiers. Give diagnostic criteria, if applicable	5&6
Data sources/measurement	8*	For each variable of interest, give sources of data and details of methods of assessment (measurement). Describe comparability of assessment methods if there is more than one group	5&6
Bias	9	Describe any efforts to address potential sources of bias	
Study size	10	Explain how the study size was arrived at	5
Quantitative variables	11	Explain how quantitative variables were handled in the analyses. If applicable, describe which groupings were chosen and why	5&6
Statistical methods	12	(*a*) Describe all statistical methods, including those used to control for confounding	6
		(*b*) Describe any methods used to examine subgroups and interactions	n/a
		(*c*) Explain how missing data were addressed	6
		(*d*) *Cohort study*—If applicable, explain how loss to follow-up was addressed<br>*Case-control study*—If applicable, explain how matching of cases and controls was addressed<br>*Cross-sectional study*—If applicable, describe analytical methods taking account of sampling strategy	n/a
		(*e*) Describe any sensitivity analyses	6

For peer review only - http://bmjopen.bmj.com/site/about/guidelines.xhtml

Section/Topic	Item No	Recommendation	Reported on Page No
**Results**			
Participants	13*	(a) Report numbers of individuals at each stage of study—eg numbers potentially eligible, examined for eligibility, confirmed eligible, included in the study, completing follow-up, and analysed	5 & 6
		(b) Give reasons for non-participation at each stage	5 & 6
		(c) Consider use of a flow diagram	n/a
Descriptive data	14*	(a) Give characteristics of study participants (eg demographic, clinical, social) and information on exposures and potential confounders	8, 9, 10
		(b) Indicate number of participants with missing data for each variable of interest	9 & 10
		(c) *Cohort study*—Summarise follow-up time (eg, average and total amount)	n/a
Outcome data	15*	*Cohort study*—Report numbers of outcome events or summary measures over time	n/a
		Case-control study—Report numbers in each exposure category, or summary measures of exposure	n/a
		Cross-sectional study—Report numbers of outcome events or summary measures	8
Main results	16	(*a*) Give unadjusted estimates and, if applicable, confounder-adjusted estimates and their precision (eg, 95% confidence interval). Make clear which confounders were adjusted for and why they were included	8, 9, 10
		(*b*) Report category boundaries when continuous variables were categorized	n/a
		(*c*) If relevant, consider translating estimates of relative risk into absolute risk for a meaningful time period	n/a
Other analyses	17	Report other analyses done—eg analyses of subgroups and interactions, and sensitivity analyses	13
**Discussion**			
Key results	18	Summarise key results with reference to study objectives	14
Limitations	19	Discuss limitations of the study, taking into account sources of potential bias or imprecision. Discuss both direction and magnitude of any potential bias	14 & 15
Interpretation	20	Give a cautious overall interpretation of results considering objectives, limitations, multiplicity of analyses, results from similar studies, and other relevant evidence	15
Generalisability	21	Discuss the generalisability (external validity) of the study results	15
**Other Information**			
Funding	22	Give the source of funding and the role of the funders for the present study and, if applicable, for the original study on which the present article is based	17

**Give information separately for cases and controls in case-control studies and, if applicable, for exposed and unexposed groups in cohort and cross-sectional studies.*

Note: An Explanation and Elaboration article discusses each checklist item and gives methodological background and published examples of transparent reporting. The STROBE checklist is best used in conjunction with this article (freely available on the Web sites of PLoS Medicine at http://www.plosmedicine.org/, Annals of Internal Medicine at http://www.annals.org/, and Epidemiology at http://www.epidem.com/). Information on the STROBE Initiative is available at www.strobe-statement.org.

For peer review only - http://bmjopen.bmj.com/site/about/guidelines.xhtml

BMJ Open

Contributions of treatment centre and patient characteristics to patient-reported experience of haemodialysis: A national cross-sectional study

Journal:	*BMJ Open*
Manuscript ID	bmjopen-2020-044984.R1
Article Type:	Original research
Date Submitted by the Author:	29-Jan-2021
Complete List of Authors:	Hawkins, Janine; University of Hertfordshire, Life and Medical Sciences<br>Smeeton, Nigel; University of Hertfordshire, Health and Social Work<br>Busby, Amanda; University of Hertfordshire School of Life and Medical Sciences, Life and Medical Sciences<br>Wellsted, David; University of Hertfordshire, Health Research Methods Unit<br>Rider, Beth; University of Hertfordshire, Life and Medical Sciences<br>Jones, Julia; University of Hertfordshire, Health and Social Work<br>Steenkamp, Retha; Renal Association<br>Stannard, Catherine; Renal Association<br>Gair, Rachel; Renal Association<br>van der Veer, Sabine N.; The University of Manchester<br>Corps, Claire; Leeds Teaching Hospitals NHS Trust<br>Farrington, Ken; Lister Hospital, Renal Unit; University of Hertfordshire, Life and Medical Sciences
**Primary Subject Heading**:	Renal medicine
Secondary Subject Heading:	Health services research, Patient-centred medicine
Keywords:	NEPHROLOGY, GENERAL MEDICINE (see Internal Medicine), Quality in health care < HEALTH SERVICES ADMINISTRATION & MANAGEMENT

SCHOLARONE™
Manuscripts

I, the Submitting Author has the right to grant and does grant on behalf of all authors of the Work (as defined in the below author licence), an exclusive licence and/or a non-exclusive licence for contributions from authors who are: i) UK Crown employees; ii) where BMJ has agreed a CC-BY licence shall apply, and/or iii) in accordance with the terms applicable for US Federal Government officers or employees acting as part of their official duties; on a worldwide, perpetual, irrevocable, royalty-free basis to BMJ Publishing Group Ltd ("BMJ") its licensees and where the relevant Journal is co-owned by BMJ to the co-owners of the Journal, to publish the Work in this journal and any other BMJ products and to exploit all rights, as set out in our licence.

The Submitting Author accepts and understands that any supply made under these terms is made by BMJ to the Submitting Author unless you are acting as an employee on behalf of your employer or a postgraduate student of an affiliated institution which is paying any applicable article publishing charge ("APC") for Open Access articles. Where the Submitting Author wishes to make the Work available on an Open Access basis (and intends to pay the relevant APC), the terms of reuse of such Open Access shall be governed by a Creative Commons licence – details of these licences and which Creative Commons licence will apply to this Work are set out in our licence referred to above.

Other than as permitted in any relevant BMJ Author's Self Archiving Policies, I confirm this Work has not been accepted for publication elsewhere, is not being considered for publication elsewhere and does not duplicate material already published. I confirm all authors consent to publication of this Work and authorise the granting of this licence.

For peer review only

Contributions of treatment centre and patient characteristics to patient-reported experience of haemodialysis: A national cross-sectional study

Author list

*, **Janine Hawkins[1],*Nigel Smeeton[2],Amanda Busby[1], David Wellsted[1], Beth Rider[1],Julia Jones[2],Retha Steenkamp[3],Catherine Stannard[3],Rachel Gair[3], Sabine N van der Veer[5,] Claire Corps[4], Ken Farrington[1,6]

*joint first author; **guarantor

Name	Job Title	Email
*Janine Hawkins[1]	Senior Research Fellow	j.hawkins3@herts.ac.uk
*Nigel Smeeton[2]	Senior Research Fellow/Social Statistician	n.smeeton@herts.ac.uk
Amanda Busby[1]	Senior Research Assistant	a.busby@herts.ac.uk
David Wellsted[1]	Reader in Health Research Methods	d.m.wellsted@herts.ac.uk
Beth Rider[1]	Research Assistant	b.rider@herts.ac.uk
Julia Jones[2]	Professor of Public Involvement and Health	j.jones26@herts.ac.uk
Retha Steenkamp[4]	Head of Operations	Retha.Steenkamp@renalregistry.nhs.uk
Catherine Stannard[4]	KQuIP Quality Improvement Programme Manager	Catherine.Stannard@renalregistry.nhs.uk
Rachel Gair[4]	KQuIP Programmes Lead	Rachel.Gair@renalregistry.nhs.uk
Sabine N van der Veer[5]	Lecturer in Health Data Sciences	sabine.vanderveer@manchester.ac.uk
Claire Corps[3]	Patient and Research Scientist	Claire.corps@nhs.net
Ken Farrington[1, 6]	Consultant Nephrologist and Head of Centre for Health Services and Clinical Research	Ken.farrington@nhs.net

[1]Centre for Health Services and Clinical Research, University of Hertfordshire, UK
[2]Centre for Research in Public Health and Community Care, University of Hertfordshire, UK
[3]Leeds Teaching Hospitals NHS Trust, Leeds, UK
[4]UK Renal Registry, University of Bristol, UK
[5]Centre for Health Informatics, Manchester Academic Health Science Centre, University of Manchester, UK
[6]E&N Herts NHS Trust, Stevenage, UK

Abstract 338

Body of text Words 3627

Tables 5

For peer review only - http://bmjopen.bmj.com/site/about/guidelines.xhtml

Abstract

Objectives To examine the relative importance of patient and centre level factors in determining self-reported experience of care in patients with advanced kidney disease treated by maintenance haemodialysis.

Design Analysis of data from a cross sectional national survey; the UK Renal Registry (UKRR) national Kidney Patient Reported Experience Measure (PREM) survey (2018). Centre level data were obtained from the UK Renal Registry report (2018).

Setting National survey of patients with advanced kidney disease receiving treatment with maintenance haemodialysis in UK renal centres in 2018.

Participants The Kidney PREM was distributed to all UK renal centres by the UKRR in May 2018. Each centre invited patients receiving outpatient treatment for kidney disease to complete the PREM. These included patients with chronic kidney disease, those receiving dialysis – both haemodialysis and peritoneal dialysis, and those with functioning kidney transplant. There were no formal inclusion/exclusion criteria.

Main outcome measures The Kidney PREM has 38 questions in 13 subscales. Responses were captured using a 7-point Likert scale (Never 1, Always 7). The primary outcome of interest was the mean PREM score calculated across all questions. Multilevel modelling was used to determine the proportion of variation of the mean PREM score across centres due to patient-related and centre-related factors.

Results There were records for 8253 haemodialysis patients (61% male, 77% White) from 69 renal centres (9 - 710 patients per centre). There was significant variation in mean PREM score across centres (5.35 to 6.53). In the multivariable analysis there was some variation in relation to both patient and centre level factors but these contributed little to explaining the overall variation. However, multilevel modelling showed that the overwhelming proportion of the explained variance (45%) was explained by variation between centres (40%), only a small proportion of which is identified by measured factors. Only 5% of the variation was related to patient-level factors.

Conclusions Centre rather than patient characteristics determine the experience of care of patients receiving haemodialysis. Further work is required to define the characteristics of the treating centre which determine patient experience.

STRENGTHS AND LIMITATIONS OF THIS STUDY

- A large sample size deriving from a national study involving all but two UK renal centres
- We employ multilevel analysis to explore the factors that inform patient experience of renal care at both patient- and centre-level and provide accurate estimates of their relative contributions.
- PREM results do not take into account the potential impact of negative non-response bias, i.e. the tendency to participate in the survey being negatively associated with problem reporting.
- Whilst distribution of patient scores is skewed, sensitivity modelling indicated that the reported models and interpretation are robust.

For peer review only - http://bmjopen.bmj.com/site/about/guidelines.xhtml

INTRODUCTION

As the survival of patients improves, attention is shifting from clinical and biomedical "hard metrics" as primary indicators of healthcare quality (1) towards gathering feedback from patients on aspects of their experience consistent with their values; including health, wellbeing, respect and dignity, quality of life (QoL), and independence (2). Patient experience of care, defined as 'patients' perception of the range of interactions they have with the healthcare system they use' (3-5) is increasingly recognised as an indicator of healthcare quality (5-7). The patient perspective has traditionally been assessed through measures of patient satisfaction (2). Patient-reported experience measures (PREMs) are emerging as a favoured metric for assessing healthcare quality (3, 7, 8). PREMs are standardised self-report tools designed to tap into specific domains of care (i.e. communication, care environment, healthcare team). Unlike satisfaction measures, patient-reported experience captures patient perspective of quality by assessing the extent to which specific care processes occur over time (3, 9) (e.g. *"In the last 12 months how often did your doctor give you the information you needed?"*). Patient experience allows providers to monitor the extent to which fundamental standards of healthcare quality have been met, through objective reporting (3, 7). In contrast, patient satisfaction measures assess the extent to which patients are *happy* with care, which is a reflection of patients' own subjective standards, expectations and affect, which is highly influenced by patient gratitude for the care received, and provides a less reliable measure of healthcare quality (3, 7, 10). PREMs aim to minimise subjective bias that arises from patient satisfaction measures (6). Nevertheless, patient experience influences patient satisfaction, demonstrating a causal link between these quality metrics (10). Measuring patient experience offers a unique understanding of how we appraise healthcare quality, by responding to the voice of those at the centre of care (7). Evaluated alongside patient safety and effectiveness, patient experience contributes to an overall holistic representation of healthcare quality (6, 7, 11).

PREMs can be used by healthcare providers, policymakers and regulators to assess provider performance and facilitate quality improvement initiatives (1). PREMs are also used to inform pay-for-performance schemes, by which services may receive funding increments in line with their performance (8, 9). Survey results allow for comparisons across providers (1) and can be made publicly available for use by patients and the general public. Patient experience is linked to a range of outcomes across healthcare populations; including clinical effectiveness, self-rated and objectively measured health outcomes, patient activation and treatment adherence (9, 12). This demonstrates the clinical utility of measuring patient-reported experience towards optimising healthcare delivery and patient outcomes. In the UK, approximately 6% of the adult population have a diagnosis of chronic kidney disease (CKD). Most have mild to moderate disease (Stages 1-3), with around 0.4% having advanced CKD (Stages 4 and 5). Renal replacement therapy (RRT) is

For peer review only - http://bmjopen.bmj.com/site/about/guidelines.xhtml

required for many patients with Stage 5 disease. Around 60,000 patients in the UK receive RRT, approximately half of whom are living with a renal transplant and the remainder receiving dialysis (13). Haemodialysis (HD) is the most prevalent dialysis modality. HD patients require long-term and burdensome treatment throughout their lifetime (6). The quality of this care can have a significant impact on these patients' wellbeing, health and quality of life (6, 14). Health-related quality of life (HRQoL) is often indicated by patients as being more important to them than survival (15).

Introduced in 2016, the UK Renal Registry (UKRR) Kidney PREM (supplementary file) is part of a national program that collects annual feedback from patients with kidney disease on their experience of NHS renal services, including those on HD. However, the implementation of PREMs in clinical practice has been relatively limited (6). As a result, responding to patient experience feedback remains a challenge for providers (16). To translate patient feedback into actionable information to drive quality improvements (6), we must be able to define which factors inform patient experience. Whether patient experience is mainly determined by patient characteristics (17, 18) or by those of the centre remains unknown (19). There is a growing body of literature addressing patient experience of care (and the factors which inform it), but few studies acknowledge the hierarchical framework of patients nested within centres. No previous studies have taken account of this framework in the haemodialysis population. The objective of the current study was to examine the relative importance of patient and centre level factors in determining self-reported experience of care in patients with advanced kidney disease treated by maintenance haemodialysis. We used clustered analysis (multilevel regression models) to examine the association between patient demographic and centre-level factors and patient experience, using secondary data from the national 2018 Kidney PREM survey. We have focused on patients receiving HD to reduce the heterogeneity of the sample and because data suggest poorer experience of care in this group (20). By using analytical methods that account for grouping within treatment centre, our study aims to provide evidence that treating centre makes a larger contribution to patient experience than patient characteristics, thereby supporting a systems perspective towards patient experience (1).

For peer review only

For peer review only - http://bmjopen.bmj.com/site/about/guidelines.xhtml

METHODS

PREM data

The validated PREM questionnaire was developed by a group of patients, clinicians and academics as a disease specific measure of patient experience. The third survey, conducted in 2018 (21), involved 71 adult renal centres across the UK, with kidney patients given the option of a paper version of the questionnaire, distributed and collected by centres, or completion online. Patient age, gender and ethnicity were recorded along with current treatment (peritoneal dialysis; haemodialysis and its location (at home, in hospital, in satellite); transplantation; or attending kidney clinics but not on dialysis or transplantation). Patients reported their use of hospital transport by answering transport-specific questions. Information regarding patient experience of care was collected from 38 questions grouped into 13 subscales consisting of between two and five questions, with a final question asking the patient to grade their overall experience of the service provided. Responses were captured using a 7-point Likert scale from Never (1) to Always (7), with additional options of Don't know and Not Applicable. The research reported here investigated only those patients receiving haemodialysis. The sample size was determined by the number of haemodialysis patients providing a completed PREM questionnaire in the 2018 survey.

Centre-level population characteristics

In addition to the patient-level survey data, the UK Renal Registry (UKRR) provides centre-level population characteristics (centre-level data). UKRR has collected centre-based data and published annual reports since 1998. Information provided for this study was from 2017 (13), the most recently available. Centre demographic variables included mean patient age, percentage male, ethnicity distribution (Asian, Black, White, Other, rather not say) and mean index of multiple deprivation. Follow-up information was obtained on the percentage of patients alive at the end of 2016 that died during 2017. Other centre-level information related to dialysis access for patients receiving renal replacement therapy, current or previous smoking, comorbidities at the start of renal replacement therapy (e.g. angina, previous myocardial infarction, diabetes), the percentage of patients who were late presenters (i.e. less than 90 days between first nephrology appointment and commencing RRT), and mean systolic and diastolic blood pressures. Mean blood test levels were provided for phosphate, bicarbonate, potassium, ferritin and urea reduction ratio. Information on transplant waiting lists and transplant rate was also obtained.

Analysis

The primary outcome of interest was the mean PREM score calculated across all 38 questions across the 13 subscales, analysed as a continuous variable, with a difference of greater than 10% (0.7) of the scale range (1 to 7) being regarded as clinically important. Where data were incomplete, scores were calculated if four

For peer review only - http://bmjopen.bmj.com/site/about/guidelines.xhtml

or fewer answers were missing out of the 33 questions with no filtering. Details of how the mean score was adjusted for missing responses is provided in the technical annex to the 2018 report (21).

The information available from the completed PREM questionnaires was used to obtain the patient-level variables, these being: age in years (17-21, 22-30, 31-40, 41-55, 56-64, 65-74, 75-84, 85+), gender (male, female, rather not say), ethnicity (Asian, Black, White, Other, rather not say), use of hospital transport (yes, no) and haemodialysis location (in-hospital, in-satellite, at home). Clinically relevant centre-level variables were selected. As the range of possible values differs between the constituent countries of the UK, for comparability, deciles for the Index of Multiple Deprivation were calculated. The strength of association between mean PREM score and prevalent mortality measures was assessed. These were the percentage of patients alive at the end of 2016 who died during 2017 (prevalent 1 year mortality unadjusted) and prevalent 1 year mortality adjusted to age 60 years (13).

A multivariable linear regression, which assumes independence of observations, was performed on the patient-level and centre-level variables that were at least 85% complete and available for each centre. Variables with a *P*-value of less than 0.1 were selected for multilevel modelling. Multilevel analyses were then performed on the mean scores by using the iterative generalised least squares method of estimation. Variance partition coefficients were calculated. These represent the proportion of total variation in an outcome that is due to differences occurring at each level; a high coefficient at centre level indicates that more of the variation in the model is due to differences between centres than between patients. Firstly, the variance partition coefficients were calculated for the null model to give raw coefficients. Then a model was fitted adjusting for patient-level characteristics to give patient adjusted coefficients. Next, both patient-level and centre-level characteristics were entered. Finally, any interactions between variables identified were added in to give fully adjusted coefficients.

Sets of models were compared using likelihood ratio tests. In order to assess whether the patients entered into the analysis were similar to those excluded due to incomplete information, the two groups were compared on age group, gender, ethnicity and location of treatment.

Sensitivity analyses were performed as part of the modelling. As patient PREM scores were negatively skewed, an exponential transformation was applied to bring the observations closer to a normal distribution. Additionally, results were compared with and without the robust estimation of errors. Snijders/Bosker R-squared values were estimated after fitting the multilevel model to determine the proportion of variance in scores attributable to centre-level and patient-level differences.

All analyses were conducted using Stata version IC/15.1

.

For peer review only - http://bmjopen.bmj.com/site/about/guidelines.xhtml

Patient and Public Involvement

The UKRR Patient Council was consulted regarding how best to share this research, as well as the Kidney PREM and other related research, with patients, medical professionals and the public in general. The study was presented at a Patient Council meeting in March 2019 and the members' views sought in particular on our dissemination plans to maximise the impact of the research on patient care.

Feedback from the Council was that there are many ways to engage with the Kidney community such as through public user group fora, patient blogs or via the Kidney Patient Associations (KPA). It was also felt that it would be beneficial to send results directly to centre staff, as well as the hospital chief executive, medical director and clinical director to ensure the findings were received at all organisational levels. In addition, the Council were keen that treatment centres were sent their own results from the PREM, highlighting how they compare to other centres, to ensure that best practice was shared across settings.

For peer review only

RESULTS

There were records for 8253 haemodialysis patients from 69 renal centres (9 to 710 patients per centre) with two centres providing no responses. Overall, reported experience of PREM was positive (mean = 5.93, standard deviation = 1.00). There was noticeable variation between centres. Mean scores were normally distributed, ranging from 5.35 to 6.53 (median 5.96, interquartile range 5.79-6.13). In addition, centres differed markedly in the proportion of patients giving a low experience score. Taking scores of less than 5 to be low, there was a ten-fold difference between centres (3.0% to 33.3%). The range of PREM scores (1.18) is far greater than 10% (0.7) of the scale range (1 to 7) and more than 1 standard deviation of the overall score, giving an indication of the impact of the centre-level variation. Table 1 shows PREM summary scores for groups based on demographic characteristics. Of note, those unwilling to disclose their gender or ethnicity (selecting “Rather Not Say”) on average gave lower experience scores. Some variation was seen across age groups, with those over 65 years reporting a better experience. However, the difference was small, ranging from 5.71 (18-21 years) to 6.01 (85+ years).

For peer review only

For peer review only - http://bmjopen.bmj.com/site/about/guidelines.xhtml

Table 1. Mean reported patient experience scores by patient characteristics in the analysed sample (percentages out of patients with the information present, *n* = 8253)

Variable	Observations	Mean	Standard deviation	Median
Age (years)				
17-21	28 (0.3%)	5.71	1.04	5.94
22-30	160 (2.0%)	5.79	1.07	6.05
31-40	325 (4.0%)	5.87	1.10	6.28
41-55	1314 (16.1%)	5.80	1.11	6.14
56-64	1540 (18.9%)	5.86	1.06	6.15
65-74	2255 (27.7%)	6.00	0.96	6.31
75-84	2046 (25.1%)	6.01	0.91	6.27
85+	477 (5.9%)	6.01	0.94	6.28
Missing	108			
Gender				
Male	4481 (60.8%)	5.96	0.98	6.26
Female	2861 (38.8%)	5.91	1.00	6.21
Not say	33 (0.4%)	4.43	1.42	4.36
Missing	878			
Ethnicity				
Asian	893 (11.5%)	5.74	1.05	5.97
Black	541 (6.9%)	5.83	1.04	6.09
White	6025 (77.3%)	5.98	0.97	6.28
Other	210 (2.7%)	5.99	0.93	6.27
Not say	129 (1.7%)	5.12	1.40	5.29
Missing	455			
Location of treatment				
Home	270 (3.4%)	6.12	0.91	6.42
Satellite	4131 (51.6%)	5.93	1.00	6.24
Hospital	3600 (45.0%)	5.93	1.01	6.22
Missing	252			
Hospital transport				
Yes	5226 (63.3%)	5.90	1.01	6.20
No	3027 (36.7%)	5.98	0.99	6.29

For peer review only

For peer review only - http://bmjopen.bmj.com/site/about/guidelines.xhtml

Missing	0

Those centre-level variables with at least 85% completeness and recorded by all renal centres are shown in Table 2. These consisted of socio-demographic variables at both patient summary and centre levels, centre size, transplanting centre (yes/no), blood test results and waiting list information. Although the data for percentage White at renal centre level were more than 85% complete, they were unavailable for 7 centres, all in Scotland, and so were excluded. However, there was a strong negative correlation between percentage White and size of centre (r = –0.7922) suggesting that centre size is a good proxy variable for percentage White. No association was found between mean PREM score and prevalent mortality, either unadjusted (r = 0.0482), or age-adjusted (r = -0.0500)

Table 2. Descriptive statistics for centres. RRT = renal replacement therapy

	N	Mean	Standard deviation	Range Across Centres
Mean Age* (years)	69	65.4	2.5	60.0 - 72.8
Percentage Male*	69	62.0	4.1	47.1 - 70.3
Mean Index of Multiple Deprivation Decile*	69	4.7	1.1	1 - 7
Transplanting Centre*				
Yes	22 (31.9%)			
No	47 (68.1%)			
Size of Centre*	69	891	685	124 – 3417
Mean Haemoglobin Level (g/L)*	69	109.2	2.6	103.4 – 116.0
Mean Phosphate Level (mmol/L)	69	1.54	0.13	0.79 - 1.74
Mean Ferritin (ng/mL)	66	492	116	297 - 889
Wait Listed within 2 years starting RRT (%)	69	40.9	11.6	14.3 - 65.4
Transplant within 2 years of waiting list (%)	69	53.4	15.3	25 – 100
Prevalent 1-year mortality unadjusted (%)	69	16.6	3.3	10.2 - 25.7
Prevalent 1-year mortality adjusted to age 60 (%)	69	12.0	2.3	6.4 – 17.3

The results from the multivariable linear regression are shown in Table 3. All patient level variables were retained in the model, along with selected centre-level variables as indicated in Table 2. Adjusting for other patient and centre-level variables increased the effect of age on the PREM scores, with a wider range of 0.53 across the age groups (compared to 0.3 as shown in Table 1). Several centre-level variables were found to be reliably associated with mean scores in the adjusted model, however the range of effect for any variable was limited (95% confidence interval range between .03, and .44).

For peer review only - http://bmjopen.bmj.com/site/about/guidelines.xhtml

For peer review only

Table 3. Results for the multivariable linear regression. (*n* = 6795, 69 centres). RRT = renal replacement therapy

Variable	Coefficient	95% confidence interval	P-value
**Patient-level**			
Age (baseline 56-64)			
17-21	-0.334	(-0.734, 0.067)	0.102
22-30	-0.058	(-0.233, 0.117)	0.515
31-40	0.028	(-0.101, 0.156)	0.673
41-55	-0.032	(-0.111, 0.047)	0.424
56-64	-	-	-
65-74	0.119	(0.050, 0.187)	0.001
75-84	0.146	(0.075, 0.217)	<0.001
85+	0.197	(0.087, 0.307)	<0.001
Gender (baseline male)			
Female	-0.030	(-0.078, 0.018)	0.223
Not say	-1.072	(-1.468, -0.677)	<0.001
Ethnicity (baseline White)			
Asian	-0.054	(-0.135, 0.027)	0.193
Black	-0.078	(-0.176, 0.020)	0.121
Other	0.125	(-0.019, 0.270)	0.089
Not say	-0.627	(-0.826, -0.429)	<0.001
Location of treatment (baseline home)			
Satellite	-0.149	(-0.282, -0.016)	0.028
Hospital	-0.219	(-0.353, -0.086)	0.001
Hospital transport	-0.120	(-0.170, -0.070)	<0.001
**Centre-level**			
Mean age (per year)	0.024	(0.009, 0.039)	0.002
Percentage male (per 10%)	-0.110	(-0.185, -0.036)	0.004
Mean Index of Multiple Deprivation (IMD) decile	-0.057	(-0.086, -0.027)	<0.001
Transplant centre	0.274	(0.200, 0.348)	<0.001
Centre size (per 100 patients)	-0.024	(-0.030, -0.018)	<0.001
Mean haemoglobin (g/L)	0.013	(0.002 0.024)	0.026
Mean phosphate (mmol/L)	0.022	(-0.197, 0.240)	0.846
Mean ferritin (ng/mL)	-0.0001	(-0.0003, 0.0001)	0.297
On waiting list< 2 years from starting RRT (per 10%)	0.075	(0.049, 0.102)	<0.001
Transplant within 2 years of waiting list (per 10%)	0.015	(-0.007, 0.037)	0.184
Unadjusted prevalent mortality (per 10%)	-0.016	(-0.108, 0.076)	0.736

Table 4 provides details of relationships between the explanatory variables at the level of centre and patient, and the mean patient experience score. Data for the multilevel modelling were obtained from 6945 (84.2%) patients, with 69 centres represented. Patients who were included differed from those

For peer review only - http://bmjopen.bmj.com/site/about/guidelines.xhtml
For peer review only

excluded in terms of ethnicity (White: 77.8% v. 72.6%) and location of treatment (hospital: 45.4% v. 42.0%). However, age (65+: 58.8% v. 57.9%), gender (male: 60.8% v. 59.5%) and use of hospital transport (yes: 62.9% v. 65.8%) were similar.

Table 4. Results for the multilevel modelling. (*n* = 6945, 69 centres). RRT = renal replacement therapy

Variable	Coefficient	95% confidence interval	P-value
**Patient-level**			
Age (baseline 56-64)			
17-21	-0.359	(-0.806, 0.089)	0.116
22-30	-0.088	(-0.287, 0.112)	0.389
31-40	0.022	(-0.098, 0.142)	0.715
41-55	-0.035	(-0.116, 0.046)	0.396
65-74	0.118	(0.047, 0.188)	0.001
75-84	0.147	(0.083, 0.212)	<0.001
85+	0.219	(0.124, 0.313)	<0.001
Gender (baseline male)			
Female	-0.027	(-0.076, 0.022)	0.275
Not say	-1.074	(-1.662, -0.485)	<0.001
Ethnicity (baseline White)			
Asian	-0.054	(-0.166, 0.057)	0.339
Black	-0.052	(-0.162, 0.058)	0.352
Other	0.122	(-0.059, 0.304)	0.186
Not say	-0.625	(-0.916, -0.334)	<0.001
Location of treatment (baseline home)			
Satellite	-0.127	(-0.258, 0.005)	0.060
Hospital	-0.231	(-0.383, -0.079)	0.003
Hospital transport	-0.128	(-0.183, -0.072)	<0.001
**Centre-level**			
Mean age (per year)	0.015	(-0.012, 0.043)	0.268
Percentage male (per 10%)	-0.122	(-0.232, -0.011)	0.031
Mean IMD decile	-0.035	(-0.086, 0.016)	0.174
Transplant centre	0.291	(0.165, 0.416)	<0.001
Centre size (per 100 patients)	-0.027	(-0.037, -0.018)	<0.001
Mean haemoglobin (g/L)	0.011	(-0.009, 0.031)	0.267
On waiting list < 2 years from starting RRT (per 10%)	0.055	(0.015, 0.095)	0.007

At the centre level, higher mean scores were associated with a higher percentage placed on the waiting list for a transplant within two years of starting renal replacement therapy. Lower scores were associated with larger centres. At the patient level, lower mean scores were associated with younger patients (< 56 years), refusal to disclose gender, refusal to disclose ethnicity, use of hospital transport and receipt of HD in-

For peer review only - http://bmjopen.bmj.com/site/about/guidelines.xhtml

centre or in-satellite (compared with dialysis at home). Most of these effects were relatively small (< 0.4 on a scale from 1 to 7), the main exceptions being refusal to disclose gender and refusal to disclose ethnicity. Patient-level variables had a similar effect on scores in the multilevel model as they did in the multivariable linear regression model (tables 3 and 4). The sensitivity analyses performed by firstly dropping robust estimation and then using the transformed data each produced similar results to those of the main analysis.

Table 5 shows the estimated variance for each model. The total variance decreases as the patient-level variables are added and decreases further once adjusted for centre-level variables (i.e. as more of the variance in patient scores was explained). The Snijders-Bosker R-squared values (22) suggest that 40.1% of variation in scores is explained by centre variation and just 5.3% of variation is accounted for by differences in patient characteristics.

Table 5: Variance at level of centre and patient, before and after adjustment for explanatory variables

Level of adjustment	Variance (total)	Variance (cons)	Variance (residual)	Variance accounted for	
				Patient level variables (%)*	Centre level variables (%)*
Unadjusted	1.0166	0.0407	0.9759		
Adjusted for patient level variables	0.9763	0.0433	0.9330		
Adjusted for patient and centre level variables	0.9486	0.0187	0.9299	5.3	40.1

For peer review only - http://bmjopen.bmj.com/site/about/guidelines.xhtml

DISCUSSION

Statement of principal findings

This study provides important insights into what influences patient experience of care in HD patients in this national sample of respondents of the UKRR/KCUK Kidney PREM 2018 survey. In this study, patient characteristics - younger patients (< 56 years), refusal to disclose gender, refusal to disclose ethnicity, use of hospital transport and receipt of treatment in centre or satellite - were only modestly associated with patient experience. These effects are small in magnitude, indicating a limited impact on patient experience. Collectively, patient characteristics account for just 5% of the observed variance in the PREM score. The only important effects were observed consistently in those patients who refused to disclose their gender or ethnicity. Previous studies have also described small variations in ratings for self-reported patient experience amongst older patients, those experiencing shortened treatments (18) and some minority ethnic populations (19).

In contrast, the centre accounts for an overwhelming proportion of the variance (40%). However, our findings indicate that while centre plays a major role in determining patient experience of care, the centre-level factors that drive patient experience are unknown. Any associations between collected centre factors and patient experience were modest and of doubtful clinical importance (i.e. <10% (0.7) of the 1 to 7 scale range) and likely to have limited impact on patient experience. For example, lower scores were associated with larger centres (-0.027 per 100 patients, p<0.001) and higher scores associated with a higher percentage of patients placed on the transplant waiting list within two years of starting RRT (0.055 per 10%, p=0.007). These findings challenge the concept that providing care focused on patient characteristics is the key determinant of patient experience. In keeping with this, Brady et al have reported variation in mean patient experience scores between groups of US facilities with different characteristics e.g. size of facility, hospital-based vs free-standing, for-profit vs non-profit. Differences were relatively small, yet still outweighed those attributed to patient characteristics (19). Factors at a group (cluster) level (for example, hospitals or GP practices) can have an influence on an individual’s outcome of interest. This is typically handled in analysis using a group variable, or multi-level models. In sample size estimation where no current estimate of the “cluster effect” is published it is generally accepted that a reasonable conservative estimate of the intra-cluster correlation (ICC) is in the order of r=0.05 (23). Often, the effect of clustering is smaller. In this paper however we present evidence that indicates that for patient experience the variation between clusters (e.g. treating centres, i.e. the centre effect) is larger by several orders of magnitude. In fact, variation attributable to the observed patient-level variables (age, ethnicity and so on) is approximately 5%, whilst the variation attributable to the centre effect is approximately 40%.

For peer review only - http://bmjopen.bmj.com/site/about/guidelines.xhtml

Strengths and limitations of the study

Our study has considerable strengths, most notably the large sample size deriving from a national study involving all but two of the UK renal centres making it more likely that the sample was representative, raising statistical power and hence the strength of the findings. The study is also the first to employ multilevel analysis to explore the factors that inform patient experience of renal care at both patient- and centre- level and provide accurate estimates of the relative contributions for factors at both these levels. It has been suggested that centre-level numbers in excess of 50 are required to prevent bias in estimates of centre-level standard errors (24). It is worth noting that the very large sample size means that a number of so-called statistically significant effects (p values <0.05) represent small differences unlikely to be of clinical importance.

The study also has some limitations. Though PREM scores were generally high, the results do not take into account the potential impact of negative non-response bias, i.e. the tendency to participate in the survey being negatively associated with problem reporting (25). Furthermore, the distribution of scores is skewed, but sensitivity modelling indicated that the reported models and interpretation are robust. More importantly, the observed variation between centres was largely unmeasured. While there have been some calls to focus on variation between centres, there is as yet little effort to understand what these factors are. Based on our study findings, the need to address this gap is urgent.

Meaning of the study: possible explanations and implications for clinicians and policymakers

These findings have implications for care delivery, suggesting that the benefits on patient experience of personalising care delivery according to patient characteristics may be far outweighed by efforts focused on reducing variation between centres as suggested by others (1, 26). However, it is unclear just what the centre characteristics are which drive this variation. Certainly, the factors examined in this study do not provide an adequate explanation. Wider systemic problems such as understaffing, lack of managerial support, and increased pressure to meet ambitious national targets can promote a culture of care delivery which is task- rather than patient-focused (27). Though these issues reflect challenges faced universally by the NHS, the centre-wide variation in patient experience observed in this study may indicate that some centres are better equipped than others to cope with increasing pressures, while others may lack the culture and mechanism to deliver the same standard of care (1).

Unanswered questions and future research

Further study is needed to determine those centre characteristics which most influence the experience of care reported by the UK haemodialysis population. The recognition of the need for 'deep cultural change'

For peer review only - http://bmjopen.bmj.com/site/about/guidelines.xhtml

(28) in promoting quality improvement is consistent with these interpretations. Additionally, further study is needed to ascertain if this is the case across other service provisions, not just in renal settings.

In summary, this large national study of patients receiving haemodialysis suggests that it is centre rather than patient characteristics which are important in shaping patient experience of care. Our findings suggest that a shift in focus from patient characteristics to the service itself is required to understand and improve patient experience of haemodialysis and potentially of many other areas of healthcare. This study provides a clear direction for future research aimed at determining the nature of the centre characteristics which drive patient experience.

For peer review only

For peer review only - http://bmjopen.bmj.com/site/about/guidelines.xhtml

Acknowledgements

Thank you to all the patients who completed the 2018 Kidney PREM, and the UK renal centres for providing data to the UK Renal Registry. We are grateful to the UK Renal Registry for supplying the data from the 2018 PREM and 2017 UK Renal Registry Report for analysis for this study, and to the UK Renal Registry Patient Council and for Kidney Care UK for their input.

Ethics Approval

The study involved secondary analysis of robustly anonymised UK Renal Registry data. The UK Renal Registry has NHS Health Research Authority research ethics approval (16/NE/0042).

Funding

This project was funded internally by the University of Hertfordshire with a cross-school collaboration research grant.

Author Contributions

Study design: KF, JH, JJ, NS, DW.

PPI: CC, JH, JJ.

Aggregation of data: AB, JH, NS, RS.

Analysis of data: AB, KF, NS, SNvdV, DW.

Interpretation of data/results: AB, CC, KF, RG, JH, JJ, BR, CS, NS, SNvdV, DW.

Drafting manuscript: AB, KF, JH, JJ, BR, NS, SNvdV, DW

Approval of final Manuscript: All

Competing Interests

All authors have completed the ICMJE uniform disclosure form at www.icmje.org/coi_disclosure.pdf and declare: no support from any organisation for the submitted work; no financial relationships with any organisations that might have an interest in the submitted work in the previous three years; no other relationships or activities that could appear to have influenced the submitted work.

Transparency Statement

The lead authors affirm that the manuscript is an honest, accurate, and transparent account of the study being reported; that no important aspects of the study have been omitted; and that any discrepancies from the study as originally planned have been explained.

For peer review only

Data Availability

Data are held by the UK Renal Registry. To apply to access Registry data see https://www.renalreg.org/about-us/working-with-us/

Copyright

The Corresponding Author has the right to grant on behalf of all authors and does grant on behalf of all authors, an exclusive licence (or non-exclusive for government employees) on a worldwide basis to the BMJ Publishing Group Ltd to permit this article (if accepted) to be published in BMJ editions and any other BMJPGL products and sublicences such use and exploit all subsidiary rights, as set out in our licence.

For peer review only

REFERENCES

1. Raleigh VS, Frosini F, Sizmur S, Graham C. Do some trusts deliver a consistently better experience for patients? An analysis of patient experience across acute care surveys in English NHS trusts. BMJ quality & safety. 2012;21(5):381-90.
2. LaVela SL, & Gallan, A. . Evaluation and measurement of patient experience. . Patient Experience Journal. 2014;1(1):28-36.
3. Cavanaugh KL. Patient Experience Assessment is a Requisite for Quality Evaluation: A Discussion of the In-Center Hemodialysis Consumer Assessment of Health Care Providers and Systems (ICH CAHPS) Survey. Seminars in dialysis. 2016;29(2):135-43.
4. Richardson MM, Paine SS, Grobert ME, Stidley CA, Gabbay E, Harford AM, et al. Satisfaction with Care of Patients on Hemodialysis. Clinical journal of the American Society of Nephrology : CJASN. 2015;10(8):1428-34.
5. Rhee CM, Brunelli SM, Subramanian L, Tentori F. Measuring patient experience in dialysis: a new paradigm of quality assessment. Journal of nephrology. 2018;31(2):231-40.
6. Aiyegbusi OL, Kyte D, Cockwell P, Anderson N, Calvert M. A patient-centred approach to measuring quality in kidney care: patient-reported outcome measures and patient-reported experience measures. Current opinion in nephrology and hypertension. 2017;26(6):442-9.
7. Black N, Jenkinson C. Measuring patients' experiences and outcomes. BMJ (Clinical research ed). 2009;339:b2495.
8. Salisbury C, Wallace M, Montgomery AA. Patients' experience and satisfaction in primary care: secondary analysis using multilevel modelling. BMJ (Clinical research ed). 2010;341:c5004.
9. Anhang Price R, Elliott MN, Zaslavsky AM, Hays RD, Lehrman WG, Rybowski L, et al. Examining the role of patient experience surveys in measuring health care quality. Medical care research and review : MCRR. 2014;71(5):522-54.
10. Kupfer JM, Bond EU. Patient satisfaction and patient-centered care: necessary but not equal. Jama. 2012;308(2):139-40.
11. Darzi L, & Johnson, A. . High quality care for all: NHS next stage review final report.: The Stationery Office.; 2008.
12. Doyle C, Lennox L, Bell D. A systematic review of evidence on the links between patient experience and clinical safety and effectiveness. BMJ open. 2013;3(1).
13. UK Renal Registry. 21st Annual Report. 2018.
14. Palmer SC, de Berardis G, Craig JC, Tong A, Tonelli M, Pellegrini F, et al. Patient satisfaction with in-centre haemodialysis care: an international survey. BMJ open. 2014;4(5):e005020.
15. Moss AH, Davison SN. How the ESRD quality incentive program could potentially improve quality of life for patients on dialysis. Clinical journal of the American Society of Nephrology : CJASN. 2015;10(5):888-93.
16. De Silva D. Measuring patient experience. The Health Foundation.; 2013.
17. van der Veer SN, Arah OA, Visserman E, Bart HA, de Keizer NF, Abu-Hanna A, et al. Exploring the relationships between patient characteristics and their dialysis care experience. Nephrology, dialysis, transplantation : official publication of the European Dialysis and Transplant Association - European Renal Association. 2012;27(11):4188-96.
18. Dad T, Tighiouart H, Lacson E, Jr., Meyer KB, Weiner DE, Richardson MM. Hemodialysis patient characteristics associated with better experience as measured by the In-center Hemodialysis Consumer Assessment of Healthcare Providers and Systems (ICH CAHPS) survey. BMC nephrology. 2018;19(1):340.
19. Brady BM, Zhao B, Niu J, Winkelmayer WC, Milstein A, Chertow GM, et al. Patient-Reported Experiences of Dialysis Care Within a National Pay-for-Performance System. JAMA internal medicine. 2018;178(10):1358-67.
20. Song MK, Gilet CA, Lin FC, MacHardy N, DeVitoDabbs AJ, Fine JP, et al. Characterizing daily life experience of patients on maintenance dialysis. Nephrology, dialysis, transplantation : official publication of the European Dialysis and Transplant Association - European Renal Association. 2011;26(11):3671-7.
21. UK Renal Registry and Kidney Care UK. Patient Reported Experience of Kidney Care in the UK. 2018 [Available from: https://www.renalreg.org/wp-content/uploads/2017/05/PREM-report-2018-final.pdf
22. Snijders TAB, & Bosker, R. J. . Multilevel analysis: an introduction to basic and advanced multilevel modelling. . London: Sage; 2012.

For peer review only - http://bmjopen.bmj.com/site/about/guidelines.xhtml

23. van Breukelen GJ, Candel MJ. Calculating sample sizes for cluster randomized trials: we can keep it simple and efficient! Journal of clinical epidemiology. 2012;65(11):1212-8.
24. Maas CJM, Hos JJ. Sufficient Sample Size for Multilevel Modelling. Methodology. 2005;1 (3):86 -92.
25. Perneger TV, Chamot E, Bovier PA. Nonresponse bias in a survey of patient perceptions of hospital care. Medical care. 2005;43(4):374-80.
26. Senn S. Mastering variation: variance components and personalised medicine. Statistics in medicine. 2016;35(7):966-77.
27. Dixon-Woods M, Baker R, Charles K, Dawson J, Jerzembek G, Martin G, et al. Culture and behaviour in the English National Health Service: overview of lessons from a large multimethod study. BMJ quality & safety. 2014;23(2):106-15.
28. High Quality Care for All: The NHS Next Stage Review Final Report. https://assets.publishing.service.gov.uk/government/uploads/system/uploads/attachment_data/file/228836/7432.pdf. Department of Health; 2008.

For peer review only

For peer review only - http://bmjopen.bmj.com/site/about/guidelines.xhtml

Kidney Care UK

NHS

The Annual Survey of Patient Reported Experience Measures (PREM)

The annual PREM survey has been designed by patients and professionals working together to find out how you feel about the services your kidney unit provides. The survey gives us feedback on renal services both locally for your unit, and nationally, and ensures that the views of kidney patients are heard. From this information, we can see what we are doing well and where we can do better. Your views matter and we act on them to help us improve services. We will provide you with feedback on the results. Information on the national results can be obtained from the UK Renal Registry or Kidney Care UK websites, along with the previous year's results.

The survey is completely anonymous, your name will not appear anywhere on the survey.

Completing the survey

The survey should take about 15 minutes to complete. Please only tick one box for each question or statement, otherwise your answer will not count.

If you prefer you can complete the survey online at www.renalreg.org/projects/prem. The online survey is available in English, Welsh, Urdu and Gujarati. Please only complete one paper PREM or one online, not both.

You can ask your partner, a friend or family member to help you complete the survey. Choosing not to take part will not affect your care in any way. When you complete the survey think about your experience of care during the last few times that you have attended. Please fill in the survey as truthfully as possible.

On completion

Please place the completed questionnaire in the envelope provided, seal it, and post it in the post box or hand it to a staff member. By completing the questionnaire you are consenting to your answers being sent to and held by the UK Renal Registry and your renal unit.

If you have any questions or concerns about the survey please contact the UK Renal Registry by emailing Catherine.stannard@renalregistry.nhs.uk or by calling 0117 414 8151.

Please complete the name of the renal/satellite unit where you are completing this survey from.

UKRR Code ☐☐☐☐☐☐☐ (To be filled in by a member of staff)

Renal Unit ☐☐☐☐☐☐☐☐☐☐☐☐☐☐☐☐☐☐☐☐

Current treatment ☐ Peritoneal dialysis ☐ Haemodialysis ☐ Transplant ☐ Attending kidney clinic but not on dialysis or transplantation

If you currently receive Haemodialysis, do you receive this…

☐ At Home ☐ In-Hospital ☐ In-Satellite

For peer review only - http://bmjopen.bmj.com/site/about/guidelines.xhtml

Please turn over the page

Age ☐ 17-21 ☐ 22-30 ☐ 31-40 ☐ 41-55
☐ 56-64 ☐ 65-74 ☐ 75-84 ☐ 85+

Gender ☐ Male ☐ Female ☐ I would rather not say

Ethnicity ☐ Asian ☐ Black ☐ White ☐ Other ☐ I would rather not say

Do you use PatientView? ☐ Yes ☐ No ☐ Don't Know

The following questions ask you about your experience with the renal unit, your kidney disease and treatment.

All the questions are answered on a scale of 1 to 7 where 1 is negative and 7 is positive.

For each question there's also a 'don't know' and 'not applicable' option.

SECTION 1: ACCESS TO THE RENAL TEAM

	**Never** 1	2	3	4	5	6	**Always** 7	Don't know	Not Applicable
1. Does the renal team take time to answer your questions about your kidney disease or treatment?	☐	☐	☐	☐	☐	☐	☐	☐	☐
2. Would you feel comfortable to contact the unit from home if you were anxious or worried?	☐	☐	☐	☐	☐	☐	☐	☐	☐
3. Would you feel able to ask for an additional appointment with your kidney doctor if you wanted to?	☐	☐	☐	☐	☐	☐	☐	☐	☐

For peer review only

 For peer review only - http://bmjopen.bmj.com/site/about/guidelines.xhtml *Please turn over the page*

SECTION 2: SUPPORT

Does the renal team help you to get the support you want with:

	Never 1	2	3	4	5	6	Always 7	Don't know	Not Applicable
4. Medical issues resulting from your kidney disease?	☐	☐	☐	☐	☐	☐	☐	☐	☐
5. Any other concerns or anxieties resulting from your kidney disease or treatment?	☐	☐	☐	☐	☐	☐	☐	☐	☐
6. Accessing patient support groups such as Kidney Patient Associations (KPA)?	☐	☐	☐	☐	☐	☐	☐	☐	☐

SECTION 3: COMMUNICATION

Do you think there is good communication between:

	Never 1	2	3	4	5	6	Always 7	Don't know	Not Applicable
7. You and your renal team?	☐	☐	☐	☐	☐	☐	☐	☐	☐
8. Members of the renal team?	☐	☐	☐	☐	☐	☐	☐	☐	☐
9. Your GP and the renal team?	☐	☐	☐	☐	☐	☐	☐	☐	☐
10. The renal team and other medical specialists?	☐	☐	☐	☐	☐	☐	☐	☐	☐
11. The renal team and other non-healthcare services if you need them, such as social work or housing?	☐	☐	☐	☐	☐	☐	☐	☐	☐

For peer review only

SECTION 4: PATIENT INFORMATION

Does the renal team:

	Never 1	2	3	4	5	6	Always 7	Don't know	Not Applicable
12. Explain things to you in a way that is easy to understand?	☐	☐	☐	☐	☐	☐	☐	☐	☐
13. Give you as much information about your kidney disease or treatment as you want?	☐	☐	☐	☐	☐	☐	☐	☐	☐

SECTION 5: FLUID INTAKE AND DIET

Thinking about the advice you are given about <u>fluid intake</u>:

	Never 1	2	3	4	5	6	Always 7	Don't know	Not Applicable
14. Does the renal team give you clear advice on your **fluid intake**?	☐	☐	☐	☐	☐	☐	☐	☐	☐

Thinking about the advice you are given about <u>diet</u>:

	Never 1	2	3	4	5	6	Always 7	Don't know	Not Applicable
15. Does the renal team give you clear advice on your **diet**?	☐	☐	☐	☐	☐	☐	☐	☐	☐

SECTION 6: NEEDLING

If you are on in-hospital or in-satellite haemodialysis please answer question 16, otherwise please go to SECTION 7: TESTS

	Never 1	2	3	4	5	6	Always 7	Don't know	Not Applicable
16. How often do the renal team insert your needles with as little pain as possible?	☐	☐	☐	☐	☐	☐	☐	☐	☐

For peer review only

For peer review only - http://bmjopen.bmj.com/site/about/guidelines.xhtml

Please turn over the page

SECTION 7: TESTS

	Never 1	2	3	4	5	6	Always 7	Don't know	Not Applicable
17. Do you understand the **reasons** for your tests?	☐	☐	☐	☐	☐	☐	☐	☐	☐
18. Do you get your test results back within an acceptable time period?	☐	☐	☐	☐	☐	☐	☐	☐	☐
19. Do you understand the **results** of your tests?	☐	☐	☐	☐	☐	☐	☐	☐	☐

SECTION 8: SHARING DECISIONS ABOUT YOUR CARE

Does the renal team:

	Never 1	2	3	4	5	6	Always 7	Don't know	Not Applicable
20. Talk with you about your treatment and life goals?	☐	☐	☐	☐	☐	☐	☐	☐	☐
21. Enable you to participate in decisions about your kidney care as much as you want?	☐	☐	☐	☐	☐	☐	☐	☐	☐
22. Talk to you about taking a more active role in managing your own kidney care?	☐	☐	☐	☐	☐	☐	☐	☐	☐

SECTION 9: PRIVACY AND DIGNITY

	Never 1	2	3	4	5	6	Always 7	Don't know	Not Applicable
23. Are you given enough privacy when discussing your condition or treatment?	☐	☐	☐	☐	☐	☐	☐	☐	☐
24. Is your dignity respected during visits and clinical examinations?	☐	☐	☐	☐	☐	☐	☐	☐	☐

Please turn over the page

For peer review only - http://bmjopen.bmj.com/site/about/guidelines.xhtml

For peer review only

SECTION 10: SCHEDULING AND PLANNING

	Never 1	2	3	4	5	6	Always 7	Don't know	Not Applicable
25. Can you change your appointment times if they are not suitable for you?									
26. Do you feel your time is used well at your appointments relating to your kidneys?									

If you are on in-hospital or in-satellite haemodialysis, please move on to SECTION 11: HOW THE RENAL TEAM TREATS YOU. If you have blood tests done at an outpatient clinic or GP surgery, please answer question 27.

	Never 1	2	3	4	5	6	Always 7	Don't know	Not Applicable
27. Are the arrangements for your blood tests convenient for you?									

SECTION 11: HOW THE RENAL TEAM TREATS YOU

Thinking about how the renal team treats you, do they:

	Never 1	2	3	4	5	6	Always 7	Don't know	Not Applicable
28. Take you seriously?									
29. Show a caring attitude towards you?									
30. Ask you about your emotional feelings?									

For peer review only - http://bmjopen.bmj.com/site/about/guidelines.xhtml
Please turn over the page

SECTION 12: TRANSPORT

If the renal unit arranges your transport, please answer these questions.
If the unit does not arrange your transport then please move on to SECTION 13: THE ENVIRONMENT.

	Never 1	2	3	4	5	6	Always 7	Don't know	Not Applicable
31. Is the vehicle provided suitable for you?	☐	☐	☐	☐	☐	☐	☐	☐	☐
32. Is the time it takes to travel between your home and the renal unit acceptable to you?	☐	☐	☐	☐	☐	☐	☐	☐	☐
33. Once your visit to the renal unit is finished and you are ready to leave, are you able to leave within less than 30 minutes?	☐	☐	☐	☐	☐	☐	☐	☐	☐

SECTION 13: THE ENVIRONMENT

When you attend the renal unit, how would you grade:

	Poor 1	2	3	4	5	6	Excellent 7	Don't know	Not Applicable
34. Accessibility (e.g., lifts, ramps, automatic doors)?	☐	☐	☐	☐	☐	☐	☐	☐	☐
35. Comfort?	☐	☐	☐	☐	☐	☐	☐	☐	☐
36. Cleanliness?	☐	☐	☐	☐	☐	☐	☐	☐	☐
37. Waiting area?	☐	☐	☐	☐	☐	☐	☐	☐	☐
38. Parking?	☐	☐	☐	☐	☐	☐	☐	☐	☐

For peer review only

Please turn over the page

SECTION 14: YOUR OVERALL EXPERIENCE

	Worst it can be 1	2	3	4	5	6	Best it can be 7
39. How well would you grade your overall experience of the service provided by your renal unit on a scale from 1 (worst it can be) to 7 (best it can be)?	☐	☐	☐	☐	☐	☐	☐

For peer review only

Thank you for completing this questionnaire.

For further information please visit www.renalreg.org/projects/prem

STROBE Statement

Checklist of items that should be included in reports of observational studies

Section/Topic	Item No	Recommendation	Reported on Page No
**Title and abstract**	1	(*a*) Indicate the study's design with a commonly used term in the title or the abstract	1
		(*b*) Provide in the abstract an informative and balanced summary of what was done and what was found	2
**Introduction**			
Background/rationale	2	Explain the scientific background and rationale for the investigation being reported	3-4
Objectives	3	State specific objectives, including any prespecified hypotheses	4
**Methods**			
Study design	4	Present key elements of study design early in the paper	5
Setting	5	Describe the setting, locations, and relevant dates, including periods of recruitment, exposure, follow-up, and data collection	5
Participants	6	(*a*) *Cohort study*—Give the eligibility criteria, and the sources and methods of selection of participants. Describe methods of follow-up<br>*Case-control study*—Give the eligibility criteria, and the sources and methods of case ascertainment and control selection. Give the rationale for the choice of cases and controls<br>*Cross-sectional study*—Give the eligibility criteria, and the sources and methods of selection of participants	5
		(*b*) *Cohort study*—For matched studies, give matching criteria and number of exposed and unexposed<br>*Case-control study*—For matched studies, give matching criteria and the number of controls per case	n/a
Variables	7	Clearly define all outcomes, exposures, predictors, potential confounders, and effect modifiers. Give diagnostic criteria, if applicable	5&6
Data sources/measurement	8*	For each variable of interest, give sources of data and details of methods of assessment (measurement). Describe comparability of assessment methods if there is more than one group	5&6
Bias	9	Describe any efforts to address potential sources of bias	
Study size	10	Explain how the study size was arrived at	5
Quantitative variables	11	Explain how quantitative variables were handled in the analyses. If applicable, describe which groupings were chosen and why	5&6
Statistical methods	12	(*a*) Describe all statistical methods, including those used to control for confounding	6
		(*b*) Describe any methods used to examine subgroups and interactions	n/a
		(*c*) Explain how missing data were addressed	6
		(*d*) *Cohort study*—If applicable, explain how loss to follow-up was addressed<br>*Case-control study*—If applicable, explain how matching of cases and controls was addressed<br>*Cross-sectional study*—If applicable, describe analytical methods taking account of sampling strategy	n/a
		(*e*) Describe any sensitivity analyses	6

For peer review only

Section/Topic	Item No	Recommendation	Reported on Page No
**Results**			
Participants	13*	(a) Report numbers of individuals at each stage of study—eg numbers potentially eligible, examined for eligibility, confirmed eligible, included in the study, completing follow-up, and analysed	5 & 6
		(b) Give reasons for non-participation at each stage	5 & 6
		(c) Consider use of a flow diagram	n/a
Descriptive data	14*	(a) Give characteristics of study participants (eg demographic, clinical, social) and information on exposures and potential confounders	8, 9, 10
		(b) Indicate number of participants with missing data for each variable of interest	9 & 10
		(c) *Cohort study*—Summarise follow-up time (eg, average and total amount)	n/a
Outcome data	15*	*Cohort study*—Report numbers of outcome events or summary measures over time	n/a
		Case-control study—Report numbers in each exposure category, or summary measures of exposure	n/a
		Cross-sectional study—Report numbers of outcome events or summary measures	8
Main results	16	(*a*) Give unadjusted estimates and, if applicable, confounder-adjusted estimates and their precision (eg, 95% confidence interval). Make clear which confounders were adjusted for and why they were included	8, 9, 10
		(*b*) Report category boundaries when continuous variables were categorized	n/a
		(*c*) If relevant, consider translating estimates of relative risk into absolute risk for a meaningful time period	n/a
Other analyses	17	Report other analyses done—eg analyses of subgroups and interactions, and sensitivity analyses	13
**Discussion**			
Key results	18	Summarise key results with reference to study objectives	14
Limitations	19	Discuss limitations of the study, taking into account sources of potential bias or imprecision. Discuss both direction and magnitude of any potential bias	14 & 15
Interpretation	20	Give a cautious overall interpretation of results considering objectives, limitations, multiplicity of analyses, results from similar studies, and other relevant evidence	15
Generalisability	21	Discuss the generalisability (external validity) of the study results	15
**Other Information**			
Funding	22	Give the source of funding and the role of the funders for the present study and, if applicable, for the original study on which the present article is based	17

**Give information separately for cases and controls in case-control studies and, if applicable, for exposed and unexposed groups in cohort and cross-sectional studies.*

Note: An Explanation and Elaboration article discusses each checklist item and gives methodological background and published examples of transparent reporting. The STROBE checklist is best used in conjunction with this article (freely available on the Web sites of PLoS Medicine at http://www.plosmedicine.org/, Annals of Internal Medicine at http://www.annals.org/, and Epidemiology at http://www.epidem.com/). Information on the STROBE Initiative is available at www.strobe-statement.org.

For peer review only - http://bmjopen.bmj.com/site/about/guidelines.xhtml
